# High-throughput identification of chromatin-opening transcription factors

**DOI:** 10.64898/2026.10.07.757445

**Published:** 2026-11-06

**Authors:** Aidan Keith, Shruti Jain, Riza M Daza, Chengxiang Qiu, Pierre-Luc Germain, Jay Shendure, Silvia Domcke

## Abstract

Cell fate transitions involve major changes to the chromatin landscape, some of which are thought to deepen the basin around a newly adopted cell state, stabilizing gene expression programs that might otherwise be transient. Transcription factors (TFs) are central to this process, binding only a subset of the genome-wide occurrences of their cognate motifs to create accessible regions that can act as cell type-specific enhancers. However, apart from a few well-studied cases, it is not clear which of the ~1,600 mammalian TFs can directly or indirectly initiate newly accessible sites, nor why only some motif occurrences gain accessibility. Here, we performed pooled CRISPR activation (CRISPRa) screens to upregulate endogenous developmental TFs in mouse embryonic stem cells (mESCs) and assess their impact on the chromatin landscape. Following a pre-screen of 240 developmental TFs for their capacity to elicit strong changes to the transcriptome, we prioritized 60 TFs for a well-powered CRISPRa multiome screen in which we jointly profiled gene expression and chromatin accessibility across ~140,000 single cells. We identify TFs that appear to initiate new chromatin accessibility on their own ("autonomous"), others whose chromatin-opening activity appears dependent on coincident binding of co-TFs heterogeneously expressed across mESCs ("opportunistic"), and yet others which appear to induce a co-TF and then act alongside it ("hierarchical"). Taken together, our findings suggest that the ability of a TF to "pioneer" chromatin opening, when it is newly expressed at physiological levels, strongly depends on the cell state and gene regulatory network it lands in. Looking forward, a comprehensive charting of such dependencies will inform how cell fate transitions are initiated in development, homeostasis and disease, and how to engineer them in silico, in vitro and in vivo.

