# Supplementary material for: High-throughput identification of chromatin-opening transcription factors
Source: bioRxiv. 2026 Oct 9:2026.10.07.757445. Preprint. [Version 1] doi: 10.64898/2026.10.07.757445 (PMC13692219; doi:10.64898/2026.10.07.757445)
Supplement: Supplementary file 1 [file NIHPP2026.10.07.757445v1-supplement-2.pdf]

bioRxiv preprint doi: https://doi.org/10.64898/2026.10.07.757445; this version posted October 9, 2026. The copyright holder for this preprint (which was not certified by peer review) is the author/funder, who has granted bioRxiv a license to display the preprint in perpetuity. It is made available under a CC-BY 4.0 International license.

SUPPLEMENTARY FIGURES

bioRxiv preprint doi: https://doi.org/10.64898/2026.10.07.757445; this version posted October 9, 2026. The copyright holder for this preprint (which was not certified by peer review) is the author/funder, who has granted bioRxiv a license to display the preprint in perpetuity. It is made available under a CC-BY 4.0 International license.

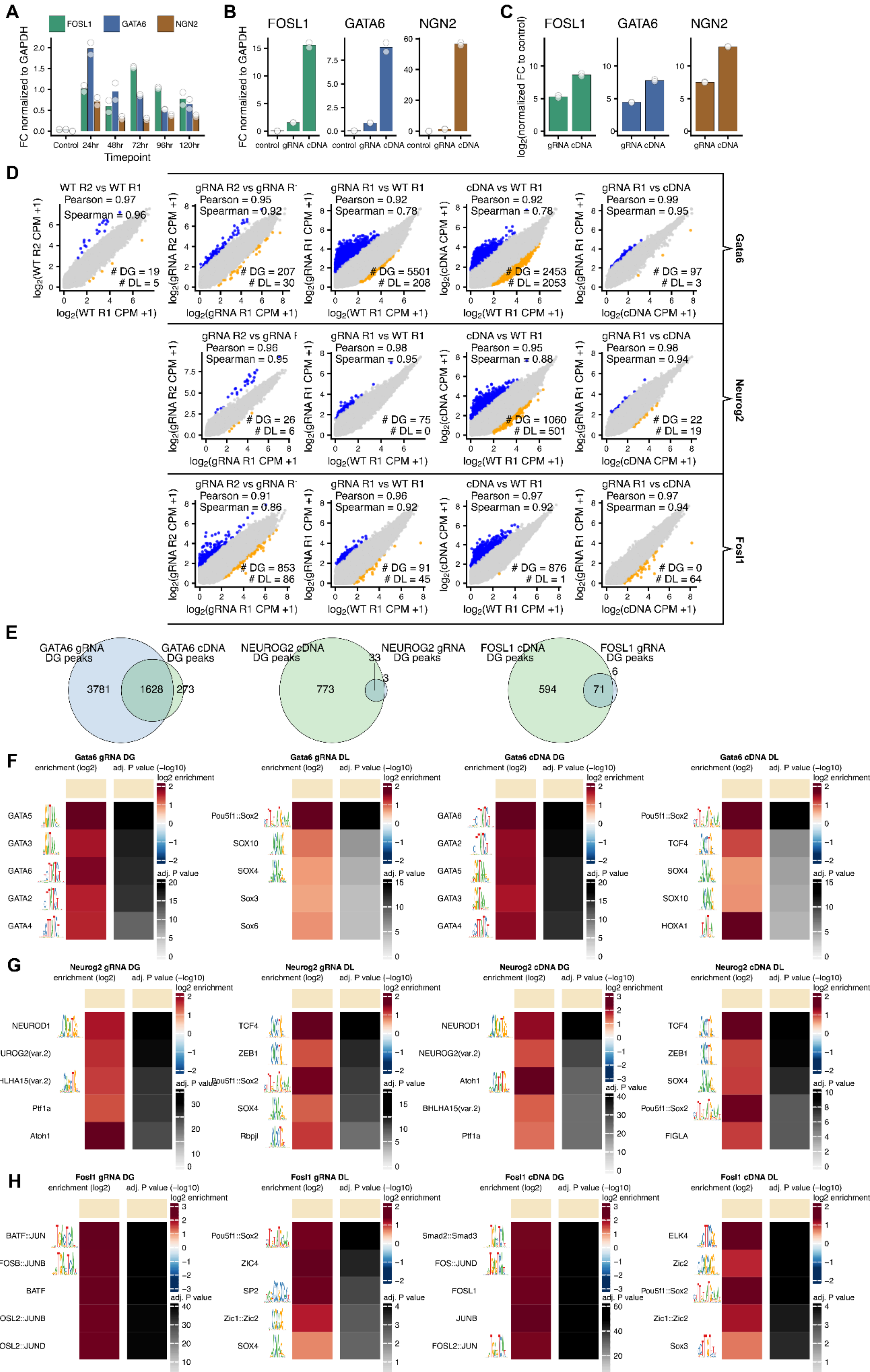

bioRxiv preprint doi: https://doi.org/10.64898/2026.10.07.757445; this version posted October 9, 2026. The copyright holder for this preprint (which was not certified by peer review) is the author/funder, who has granted bioRxiv a license to display the preprint in perpetuity. It is made available under a CC-BY 4.0 International license.

Figure S1. Comparison of TF activation and chromatin accessibility responses to CRISPRa and cDNA overexpression

(A) qPCR time series of CRISPRa activation levels from cells profiled 24, 48, 72, 96 and 120 hrs after transduction of targeting guides or mock transduced control cells. Values are normalized to *Gapdh* expression in the same sample for each of two biological replicates. **(B)** qPCR target activation levels of mock, gRNA construct, and cDNA constructs relative to *Gapdh* for *Fosl1*, *Gata6*, and *Ngn2* profiled 72 hrs after transduction. Values are normalized to *Gapdh* expression in the same sample for each of two biological replicates. **(C)** Same as the above only after normalizing activation levels to mock transductions. **(D)** Correlation plots comparing bulk chromatin accessibility ($\log_2$ CPM + 1) across replicates (R1, R2) and conditions (CRISPRa/gRNA or cDNA) for activation of *Gata6* (top row), *Neurog2* (middle row), and *Fosl1* (bottom row) across merged peaks, with each point representing a single peak. Colors denote peaks classified as differentially accessible: since cDNA experiments do not have multiple replicates, for this analysis DG and DL sites were simply defined based on their FC in accessibility (DG; $\geq \log_2(3)$ and DL; $\leq -\log_2(3)$ decrease), relative to the first sample in the comparison. Pearson and Spearman correlation coefficients are shown for each comparison. Overall there is good agreement between CRISPRa replicates (1st column) as well as CRISPRa and cDNA activation (4th column), with gained sites detected in both conditions compared to WT (2nd, 3rd column). **(E)** Venn diagram showing overlap between DG peaks in bulk *Gata6* gRNA and cDNA samples (left-most diagram), bulk *Neurog2* gRNA and cDNA samples (middle diagram), and bulk *Fosl1* gRNA and cDNA samples (right-most diagram). Peaks were defined as differentially accessible if they exhibited at least a 3-fold increase in normalized accessibility (CPM) relative to WT control bulk ATAC samples (replicate counts averaged). For CRISPRa/gRNA samples peaks were called individually for both replicates and merged, whereas for cDNA samples only one replicate was available. **(F)** Motif enrichment analysis of bulk *Gata6* gRNA vs WT DG sites (left-most heatmap), *Gata6* gRNA vs WT DL sites (second heatmap), *Gata6* cDNA vs WT DG sites (third heatmap), and *Gata6* cDNA vs WT DL sites (right-most heatmap) using monaLisa. DG and DL sites were defined as the top 200 peaks ranked by fold-change (FC) in accessibility relative to WT control (highest FC for DG, lowest FC for DL), without applying a significance or minimum FC threshold. DNA sequences centered on the peak midpoint (±250 bp) were compared to a GC-matched background sampled from the genome. The top five enriched motifs are displayed based on statistical significance ($-\log_{10}$ adjusted p-value). **(G)** Motif enrichment analysis of bulk *Neurog2* gRNA vs WT DG sites (left-most heatmap), *Neurog2* gRNA vs WT DL sites (second heatmap), *Neurog2* cDNA vs WT DG sites (third heatmap), and *Neurog2* cDNA vs WT DL sites (right-most heatmap) using monaLisa. Analysis was performed as in F. **(H)** Motif enrichment analysis of bulk *Fosl1* gRNA vs WT DG sites (left-most heatmap), *Fosl1* gRNA vs WT DL sites (second heatmap), *Fosl1* cDNA vs WT DG sites (third heatmap), and *Fosl1* cDNA vs WT DL sites (right-most heatmap) using monaLisa. Analysis was performed as in **F.**

bioRxiv preprint doi: https://doi.org/10.64898/2026.10.07.757445; this version posted October 9, 2026. The copyright holder for this preprint (which was not certified by peer review) is the author/funder, who has granted bioRxiv a license to display the preprint in perpetuity. It is made available under a CC-BY 4.0 International license.

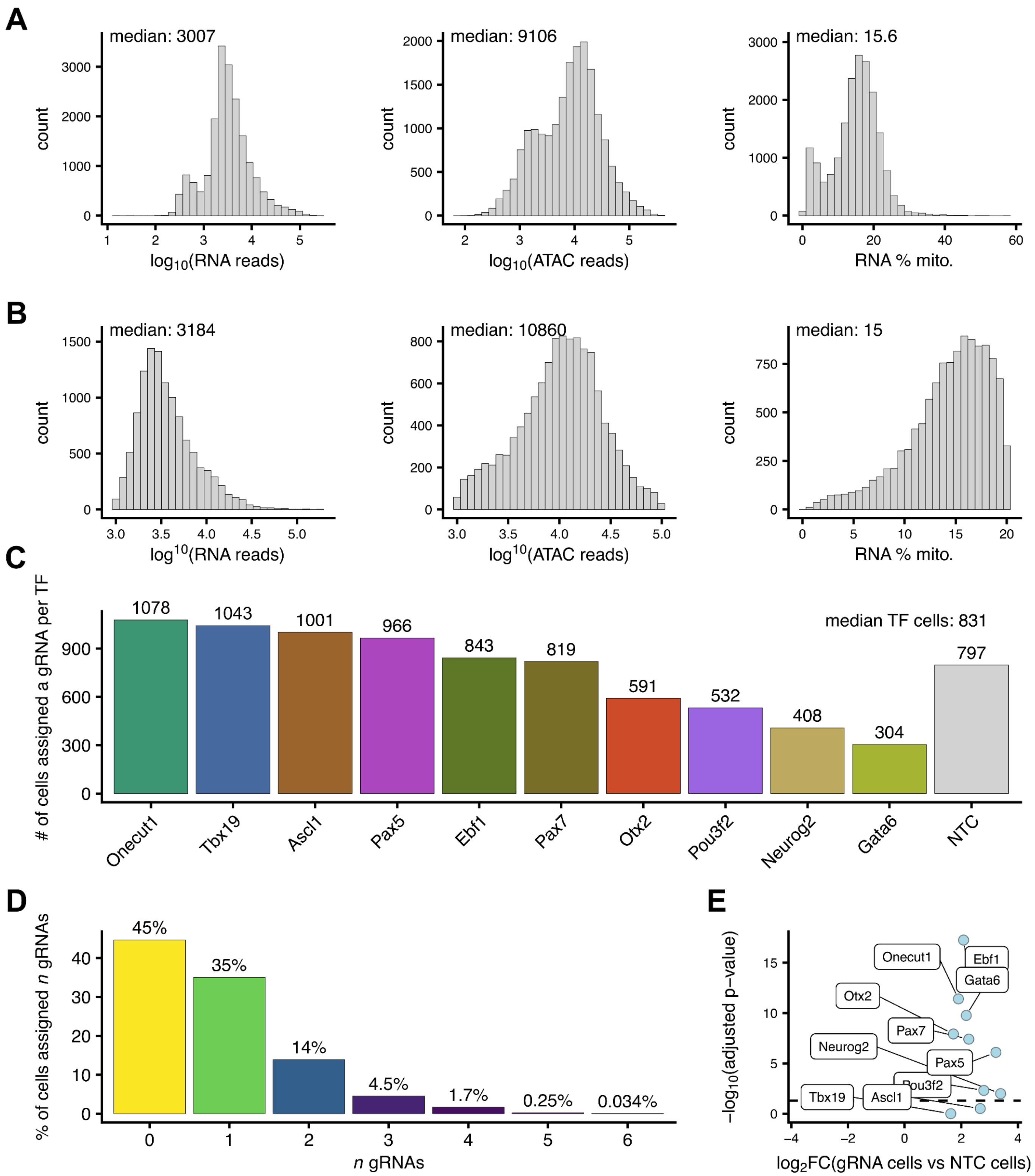

Figure S2. Pilot multiome profiling of cells with pooled CRISPRa activated TFs.

(A) From left-to-right, distributions of per-cell RNA UMIs, unique ATAC reads, and RNA mitochondrial read percentages before filtering for cells with mitochondrial read percentages ≤20% as well as RNA and ATAC counts ≥ 1,000. **(B)** Same as in **A** only after filtering for cells with mitochondrial read percentages ≤20% as well as RNA and ATAC counts ≥ 1,000 (n = 11,615 cells). **(C)** Bar plots indicating the number of cells assigned a gRNA for each targeted TF (including cells with multiple gRNAs assigned) as well as the number of cells assigned an NTC gRNA (single gRNA assignments only). **(D)**

bioRxiv preprint doi: https://doi.org/10.64898/2026.10.07.757445; this version posted October 9, 2026. The copyright holder for this preprint (which was not certified by peer review) is the author/funder, who has granted bioRxiv a license to display the preprint in perpetuity. It is made available under a CC-BY 4.0 International license.

Bar plots showing the percentage of cells assigned no gRNA, a single gRNA, or multiple gRNAs. **(E)** The fold change in expression of each target TF in cells with a corresponding gRNA for that TF versus NTC gRNA(+) cells.

bioRxiv preprint doi: https://doi.org/10.64898/2026.10.07.757445; this version posted October 9, 2026. The copyright holder for this preprint (which was not certified by peer review) is the author/funder, who has granted bioRxiv a license to display the preprint in perpetuity. It is made available under a CC-BY 4.0 International license.

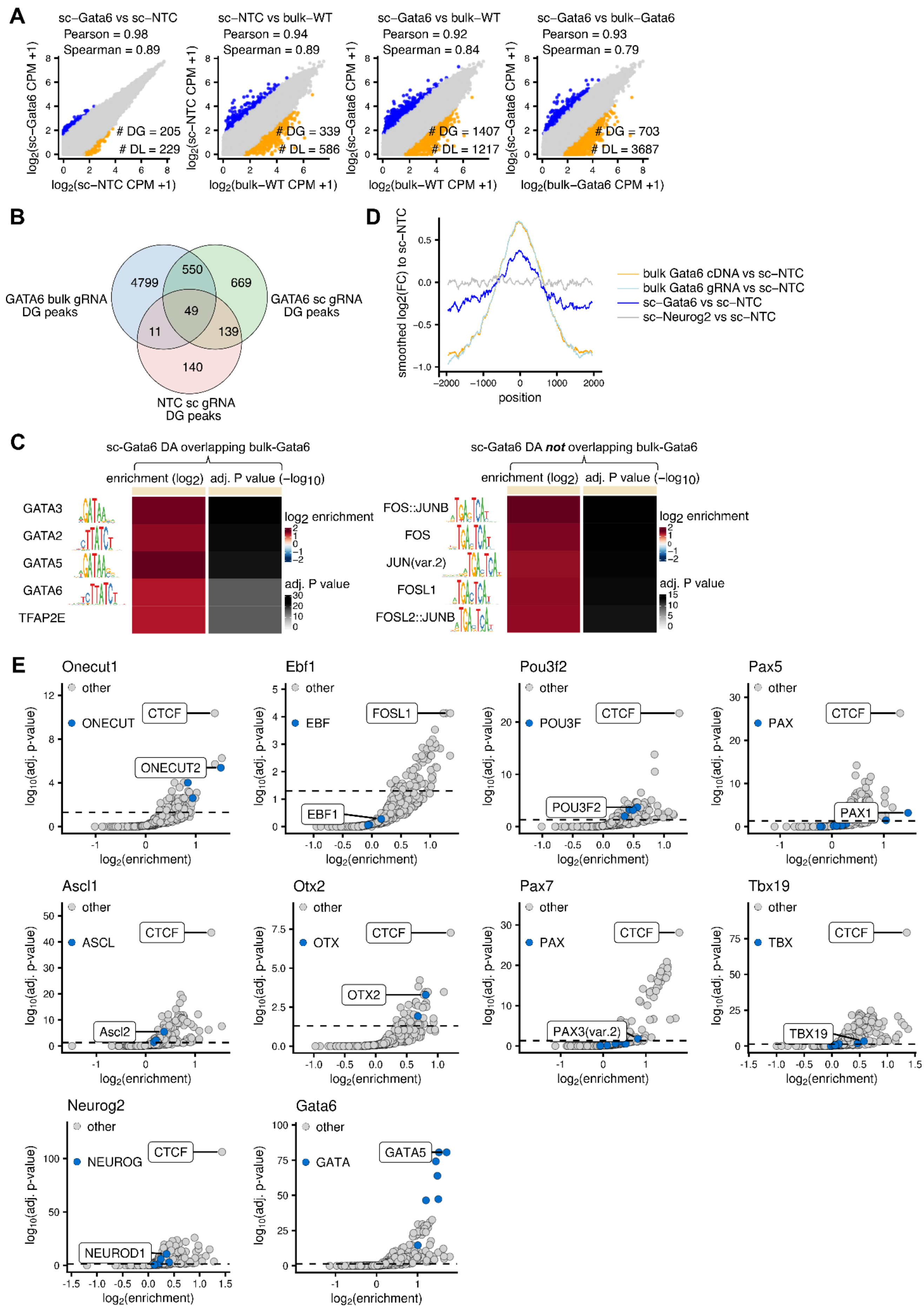

bioRxiv preprint doi: https://doi.org/10.64898/2026.10.07.757445; this version posted October 9, 2026. The copyright holder for this preprint (which was not certified by peer review) is the author/funder, who has granted bioRxiv a license to display the preprint in perpetuity. It is made available under a CC-BY 4.0 International license.

Figure S3. Shared signal between single-cell multiome profiling and bulk ATAC-seq for CRISPRa activation of *Gata6*.

(A) Correlation plots comparing chromatin accessibility ($\log_2$ CPM + 1) across pseudobulked *Gata6* gRNA(+) cells from the pilot multiome, pseudobulked NTC gRNA(+) cells from the pilot multiome, and bulk ATAC-seq data for WT cells and *Gata6* CRISPRa activated cells. Each point represents a single peak; colors denote peaks classified as differentially accessible: since not all conditions have multiple replicates, for this analysis DG and DL sites were simply defined based on their FC in accessibility (DG; ≥ $\log_2(3)$ and DL; ≤ $-\log_2(3)$ decrease), relative to the first sample in the comparison. Pearson and Spearman correlation coefficients are shown for each comparison. **(B)** Venn diagram showing overlap between the DG peaks shown in (A) for 1) pseudobulked *Gata6* gRNA(+) cells from the pilot multiome 2) pseudobulked NTC gRNA(+) cells from the pilot multiome and 3) bulk ATAC *Gata6* gRNA samples. **(C)** Motif enrichment analysis of DG peaks in the pseudobulked *Gata6* gRNA(+) cells from the pilot multiome that overlapped peaks from bulk *Gata6* gRNA ATAC samples (left heatmap) as well as DG peaks from pseudobulked *Gata6* gRNA(+) cells from the pilot multiome that did not overlap DG peaks from bulk *Gata6* gRNA ATAC samples. The top five enriched motifs are displayed based on statistical significance ($-\log_{10}$ adjusted p-value). **(D)** Chromatin accessibility centered on DG sites called in bulk *Gata6* gRNA samples, showing the smoothed fold change in normalized accessibility for bulk ATAC or single cell multiome assays relative to single cell NTC cells. Profiles are shown for bulk *Gata6* cDNA, bulk *Gata6* gRNA, single cell *Gata6* gRNA, and single cell *Neurog2* gRNA conditions. Signals were averaged across all DG sites, normalized per region and per million mapped reads, and smoothed using a rolling mean to highlight spatial trends in accessibility changes around peak centers. Whereas there is a gain in accessibility for all *Gata6* samples, whether bulk or single cell, there is none for *Neurog2* gRNA(+) cells from the same multiome pilot, as expected. **(E)** For each of the 10 TF's targeted in the pilot multiome screen, cells with detected expression of the TF transcript (≥1 UMI, RNA assay) were compared to non-targeting control (NTC) cells for differential accessibility testing on the ATAC assay (Wilcoxon rank-sum test). DG sites were defined as peaks with nominal p-value < 0.01, avg. $\log_2$ fold-change > 2, and detected in > 3% of TF-expressing cells (pct.1 > 0.03), and restricted to standard chromosomes. Motif enrichment at DG sites was performed using all vertebrate motifs from the JASPAR2020 database against a GC-matched background of randomly sampled genomic sequences. Sites were resized to a fixed 500 bp window centered on the peak midpoint prior to sequence extraction. Volcano plots of motif enrichment ($\log_2$ enrichment vs. $-\log_{10}$ adjusted p-value) are shown for each TF perturbation. Points are colored by motif identity: motifs matching the targeted TF (and, where noted, closely related family members) are shown in blue; all other motifs are shown in grey. Dashed line indicates adjusted p-value = 0.05. The top-ranked motif overall and the top-ranked TF-specific (blue) motif, by combined enrichment/significance score, are labeled in each panel.

bioRxiv preprint doi: https://doi.org/10.64898/2026.10.07.757445; this version posted October 9, 2026. The copyright holder for this preprint (which was not certified by peer review) is the author/funder, who has granted bioRxiv a license to display the preprint in perpetuity. It is made available under a CC-BY 4.0 International license.

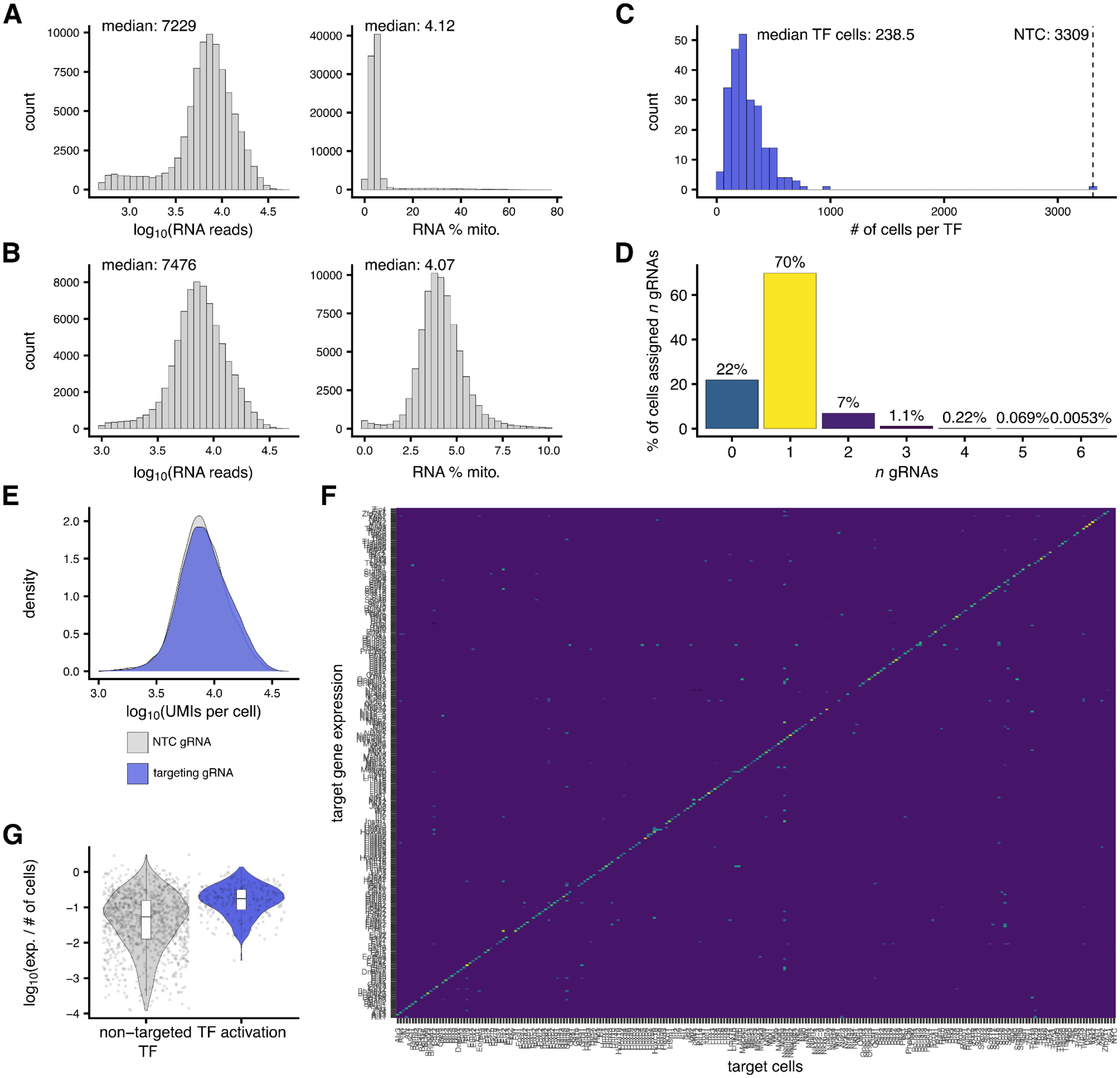

Figure S4. TF activation to physiological expression levels in 240 TF scRNA-seq screen.
(A) From left-to-right, distributions of per-cell RNA UMIs and RNA mitochondrial read percentages before filtering. **(B)** Same distributions as shown in **A** only after filtering for cells with mitochondrial read percentages ≤10% as well as RNA counts ≥ 1,000 (n = 75,340 cells). **(C)** Histogram showing the number of cells assigned a gRNA for each targeted TF (including cells with multiple gRNAs assigned) as well as the number of cells assigned an NTC gRNA (single gRNA assignments only). **(D)** Bar plots showing the percentage of cells assigned no gRNA, a single gRNA, or multiple gRNAs. **(E)** Density plots showing the per cell UMI distributions in cells assigned an NTC gRNA (grey density plot) vs cells assigned a targeting gRNA (blue density plot), to confirm that activation levels are not driven by systematic differences in control cells. **(F)** Heatmap showing the fold-change in expression of all 240 TFs (rows) in cells assigned a gRNA for each of the 240 TFs (columns) relative to NTC control cells. The right-most column shows the fold-change in

bioRxiv preprint doi: https://doi.org/10.64898/2026.10.07.757445; this version posted October 9, 2026. The copyright holder for this preprint (which was not certified by peer review) is the author/funder, who has granted bioRxiv a license to display the preprint in perpetuity. It is made available under a CC-BY 4.0 International license.

expression of all 240 TFs in NTC control cells relative to cells not assigned a gRNA. Values failing significance (adjusted p-value ≥ 0.05) were set to zero. **(G)** Violin plots showing the expression of TF genes normalized to cell count for each targeted TF in cells assigned a corresponding gRNA for that TF (blue violin, right), or the expression of all other murine TFs in NTC cells (grey violin, left). White box plots indicate the median and interquartile range.

bioRxiv preprint doi: https://doi.org/10.64898/2026.10.07.757445; this version posted October 9, 2026. The copyright holder for this preprint (which was not certified by peer review) is the author/funder, who has granted bioRxiv a license to display the preprint in perpetuity. It is made available under a CC-BY 4.0 International license.

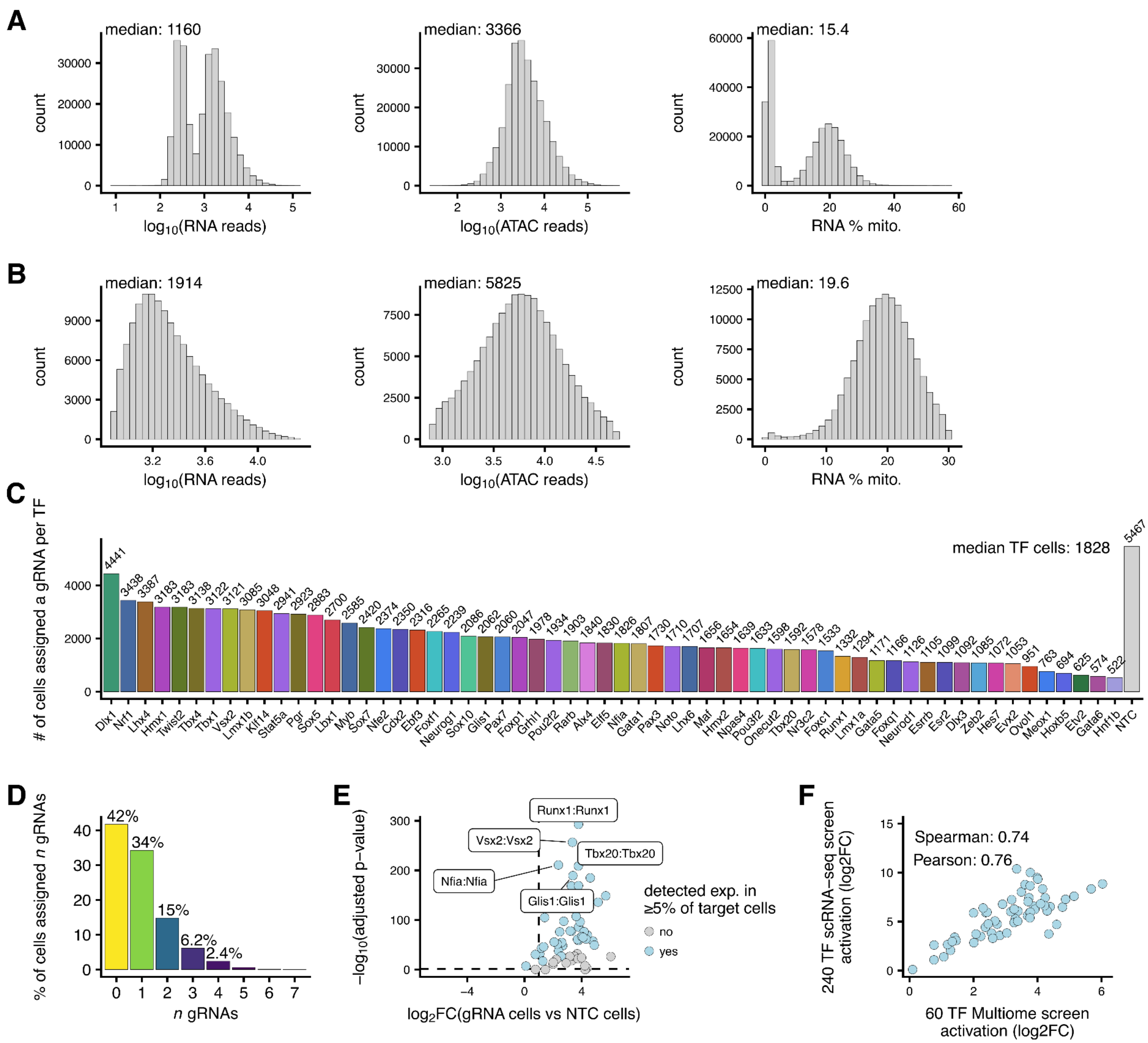

Figure S5. Cell filtering and gRNA capture for 60 TF single cell multiome screen.
(A) From left-to-right, distributions of per-cell RNA UMIs, unique ATAC reads, and RNA mitochondrial read percentages before filtering cells. (**B**) Same as in **A** only after filtering for cells with mitochondrial read percentages ≤30% as well as RNA and ATAC counts ≥ 800, ATAC counts < 50,000 & RNA counts < 20,000 (n = 139,374 cells post filtering) **(C)** Bar plots indicating the number of cells assigned a gRNA for each targeted TF (including cells with multiple gRNAs assigned) as well as the number of cells assigned an NTC gRNA (single gRNA assignments only). **(D)** Bar plots showing the percentage of cells assigned no gRNA, a single gRNA, or multiple gRNAs. **(E)** The fold change in expression of each target TF in cells with a corresponding gRNA for that TF versus NTC gRNA(+) cells. Color fill indicates whether or not expression of the corresponding TF was detected in at least 5% of the gRNA(+) cells for that TF. **(F)** Correlations in target TF activation levels (log_2FC gRNA(+) cells relative to NTC cells) from the 240-TF scRNA-seq screen (y-axis) vs the 60-TF Multiome screen (x-axis).

bioRxiv preprint doi: https://doi.org/10.64898/2026.10.07.757445; this version posted October 9, 2026. The copyright holder for this preprint (which was not certified by peer review) is the author/funder, who has granted bioRxiv a license to display the preprint in perpetuity. It is made available under a CC-BY 4.0 International license.

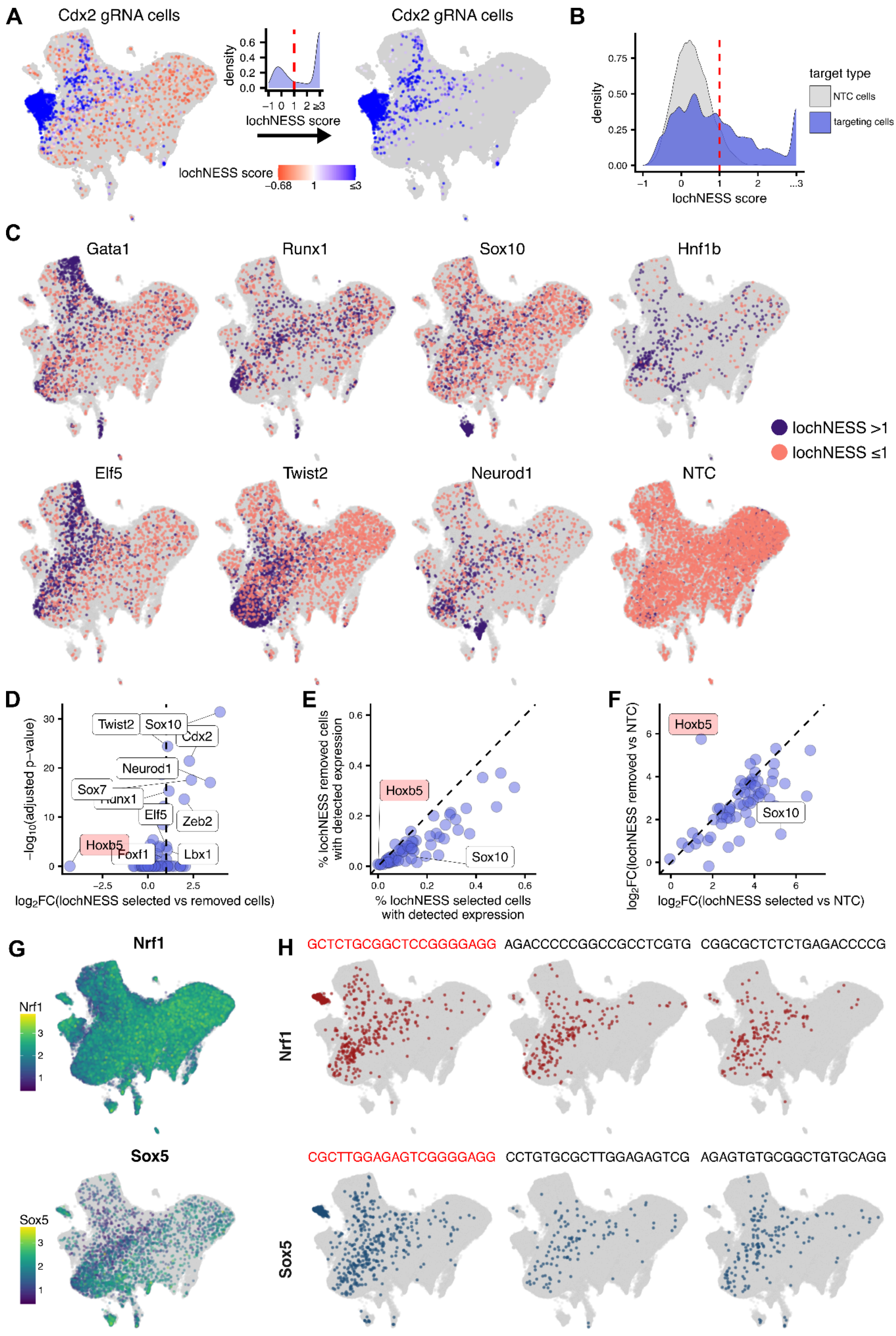

bioRxiv preprint doi: https://doi.org/10.64898/2026.10.07.757445; this version posted October 9, 2026. The copyright holder for this preprint (which was not certified by peer review) is the author/funder, who has granted bioRxiv a license to display the preprint in perpetuity. It is made available under a CC-BY 4.0 International license.

Figure S6. LochNESS filtering selects for more efficiently perturbed cells.
(A) ATAC UMAP showing lochNESS mutant scores for *Cdx2* gRNA(+) cells before (left) and after (right) applying a lochNESS cutoff of 1. All cells without a *Cdx2* gRNA are shown in light grey, while cells assigned a *Cdx2* gRNA are colored by their lochNESS score. **(B)** Density distribution of lochNESS scores for targeting gRNA(+) cells (purple) and non-targeting control (NTC) gRNA(+) cells (grey). For each TF, lochNESS scores were computed for TF-targeted cells and combined into a single density plot. NTC gRNA(+) cells were analyzed separately and plotted as their own distribution. Scores greater than 3 were capped at 3 for visualization, and a dashed vertical line marks the lochNESS cutoff of 1. **(C)** ATAC UMAPs for several gRNAs targeting TFs (plus NTC control) illustrating focal enrichments of gRNA(+) cells with higher lochNESS scores. Points in purple are gRNA(+) cells retained by lochNESS (score > 1), points in red are gRNA(+) cells removed by lochNESS (score ≤ 1), and light grey are all other cells. **(D)** Volcano plot of differential expression for the target TF between lochNESS-selected gRNA(+) cells for that TF (score > 1) vs lochNESS-removed cells for that TF (score ≤ 1), illustrating higher target TF expression in lochNESS-selected cells. The x-axis shows the $\log_2$ fold change in expression, and the y-axis shows $-\log_{10}$(adjusted p-value) from Wilcoxon tests. A dashed vertical line marks $\log_2FC = 1$. The top 10 significantly up-regulated TFs ($\log_2FC > 1$, adjusted $p < 0.05$) are labeled. *Hoxb5* appears as a prominent outlier with a negative fold change, likely reflecting stochastic differences arising from the extremely low fraction of cells with detectable *Hoxb5* expression in both lochNESS-selected and lochNESS-removed populations (see **E**). **(E)** Scatter plot comparing, for each TF, the percentage of lochNESS-selected gRNA(+) cells (x-axis) and lochNESS-removed gRNA(+) cells (y-axis) with detected target TF expression, illustrating higher detection rates in lochNESS-selected cells. Each point represents one TF. A dashed diagonal marks x = y, showing where points would fall if detection were equal. **(F)** Scatter plot showing $\log_2$ fold-change in target TF expression vs NTC for lochNESS-selected cells (x-axis) and lochNESS-removed cells (y-axis), illustrating stronger fold-changes after lochNESS selection. Each point is one TF. Dashed diagonal (x = y) shows where points would fall if activation were equal between selected and removed groups. Labels mark the TFs with the largest positive and most negative outliers i.e. $\Delta(\log_2(\text{FC selected vs NTC}) - \log_2(\text{FC removed vs NTC}))$. *Hoxb5* appears as a prominent outlier, likely reflecting stochastic differences arising from the extremely low fraction of cells with detectable *Hoxb5* expression in both lochNESS-selected and lochNESS-removed populations (see **E**). **(G)** Expression distributions of *Nrf1* (top) and *Sox5* (bottom) in the ATAC UMAP embedding. **(H)** ATAC UMAP distributions of cells assigned each of the three *Nrf1*-targeting gRNAs (top row) and each of the three *Sox5*-targeting gRNAs (bottom row). For each gRNA, colored points indicate cells assigned that gRNA that passed target-level lochNESS filtering (raw mutant score >1); all other cells are shown in gray. The gRNA(s) highlighted in red have sequence similarity to each other and show a distinct distribution in UMAP space.

bioRxiv preprint doi: https://doi.org/10.64898/2026.10.07.757445; this version posted October 9, 2026. The copyright holder for this preprint (which was not certified by peer review) is the author/funder, who has granted bioRxiv a license to display the preprint in perpetuity. It is made available under a CC-BY 4.0 International license.

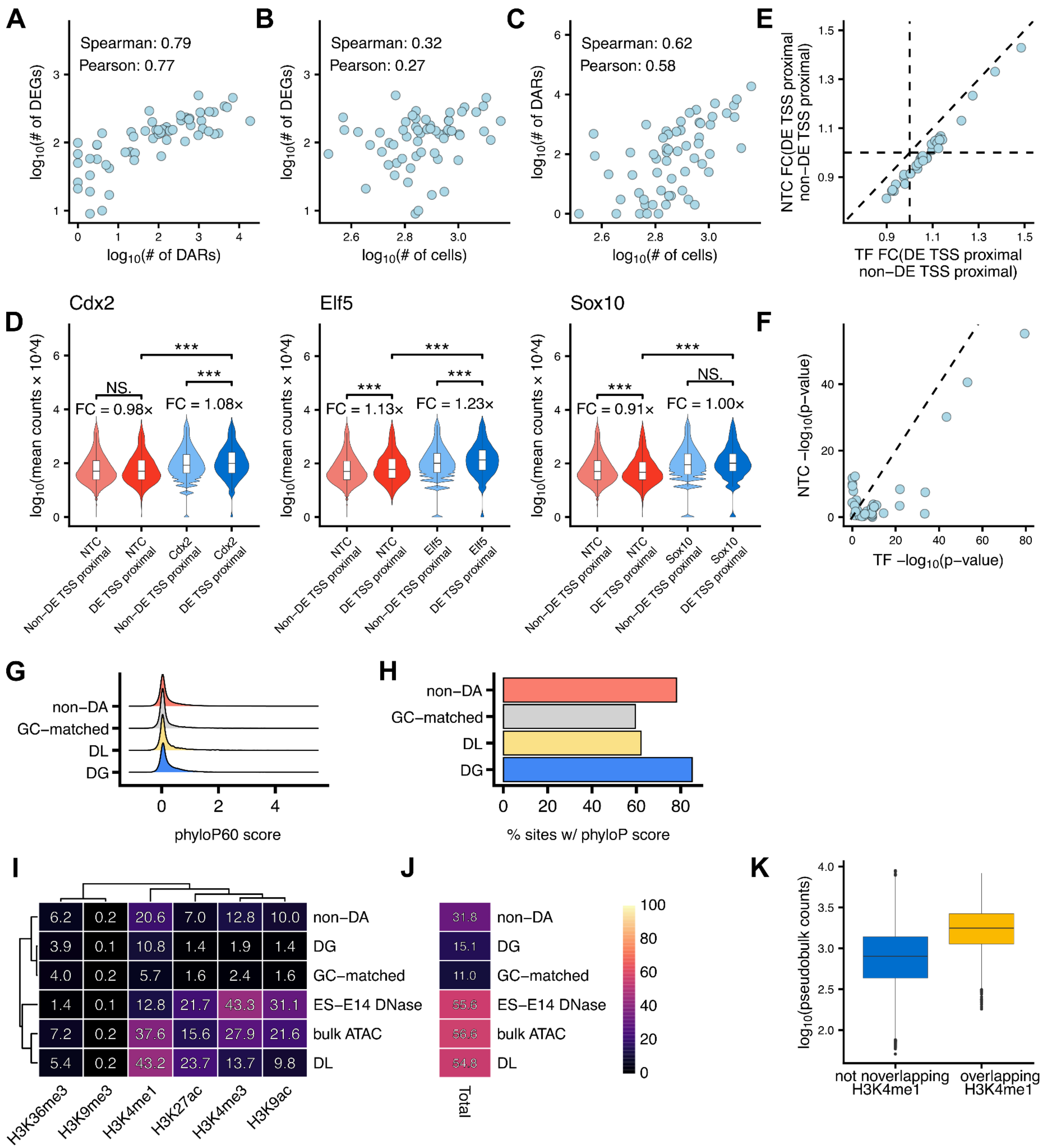

Figure S7. Characterizing differentially accessible regions (DARs) and overlap with histone modifications in mESCs

(A) Scatter plot showing the correlation between the number of DARs and the number of DEGs across TFs. **(B)** Scatter plot showing the correlation between the number of cells assigned to each TF and the number of DEGs detected for

bioRxiv preprint doi: https://doi.org/10.64898/2026.10.07.757445; this version posted October 9, 2026. The copyright holder for this preprint (which was not certified by peer review) is the author/funder, who has granted bioRxiv a license to display the preprint in perpetuity. It is made available under a CC-BY 4.0 International license.

that TF. **(C)** Scatter plot showing the correlation between the number of cells assigned to each TF and the number of DARs called for that TF. **(D)** Increased accessibility at peaks near differentially expressed genes in TF targeted cells. Violin plots show per-peak pseudobulk accessibility (mean ATAC counts per cell; plotted as $\log_{10}$(mean counts × 10^4 + 1)) for peaks within ±100 kb of the TSS of genes differentially expressed by the indicated TF ("DE TSS proximal") compared to peaks within ±100 kb of the TSS of all other genes with detected expression ("Non-DE TSS proximal"). Values are shown for NTC cells (red) and lochNESS-selected TF cells (blue) for several representative TFs. Asterisks indicate Wilcoxon rank-sum test significance for the indicated comparisons. **(E)** Scatter plot showing for each TF, the fold change (FC) in mean per-peak ATAC accessibility (mean counts per cell) for peaks within ±100 kb of TSSs of DE genes relative to peaks within ±100 kb of TSSs of non-DE genes. FC values are shown for NTC cells (y-axis) and for lochNESS-selected TF cells (x-axis). **(F)** Same as in **E**, only showing the $-\log_{10}$ p-values (Wilcoxon rank-sum test) of the fold-change comparisons. **(G)** Peaks were categorized as differentially gained (DG), differentially lost (DL), or non-differentially accessible (non-DA), and an additional background set of random genomic regions was generated from mm10, matched to DG regions by size and GC composition. Density plots show the distributions of phyloP scores for each of these categories among peaks that had a phyloP score. **(H)** Bar plots showing the percentage of sites for each category defined in **G** that had a phyloP score. **(I)** The same peak categories as defined in **G** and also including ENCODE peaks from ES-E14 DNase-seq data and bulk ATAC peaks called on our WT mESC CRISPRa cell line, which were included as additional categories for reference. The heatmap displays the fraction of peaks in each category (y-axis) that overlap ENCODE ChIP-seq peaks generated in serum-cultured E14 mESC for each of the indicated histone modifications (x-axis). Differences in cell line or culture conditions (2i + LIF in the current dataset versus serum + LIF in the ENCODE datasets) and/or shallower sequencing depth of the ChIP-seq datasets could contribute to lower overlap percentages. In addition this DNAse dataset is quite shallow (62,535 peaks) contributing to outgroup clustering.**(J)** Heatmap showing for each peak category the total fraction of peaks overlapping one or more ENCODE ChIP-seq peaks across the set of histone marks analyzed. **(K)** Boxplots showing $\log_{10}$-transformed total fragment counts for DG peaks stratified by sites overlapping H3K4me1 ENCODE peaks in WT mESCs (10.9%, n = 3,011) and sites not overlapping H3K4me1 peaks (89.1%, n = 24,731), indicating a subset DG sites might already display some accessibility/activity in the unperturbed state.

bioRxiv preprint doi: https://doi.org/10.64898/2026.10.07.757445; this version posted October 9, 2026. The copyright holder for this preprint (which was not certified by peer review) is the author/funder, who has granted bioRxiv a license to display the preprint in perpetuity. It is made available under a CC-BY 4.0 International license.

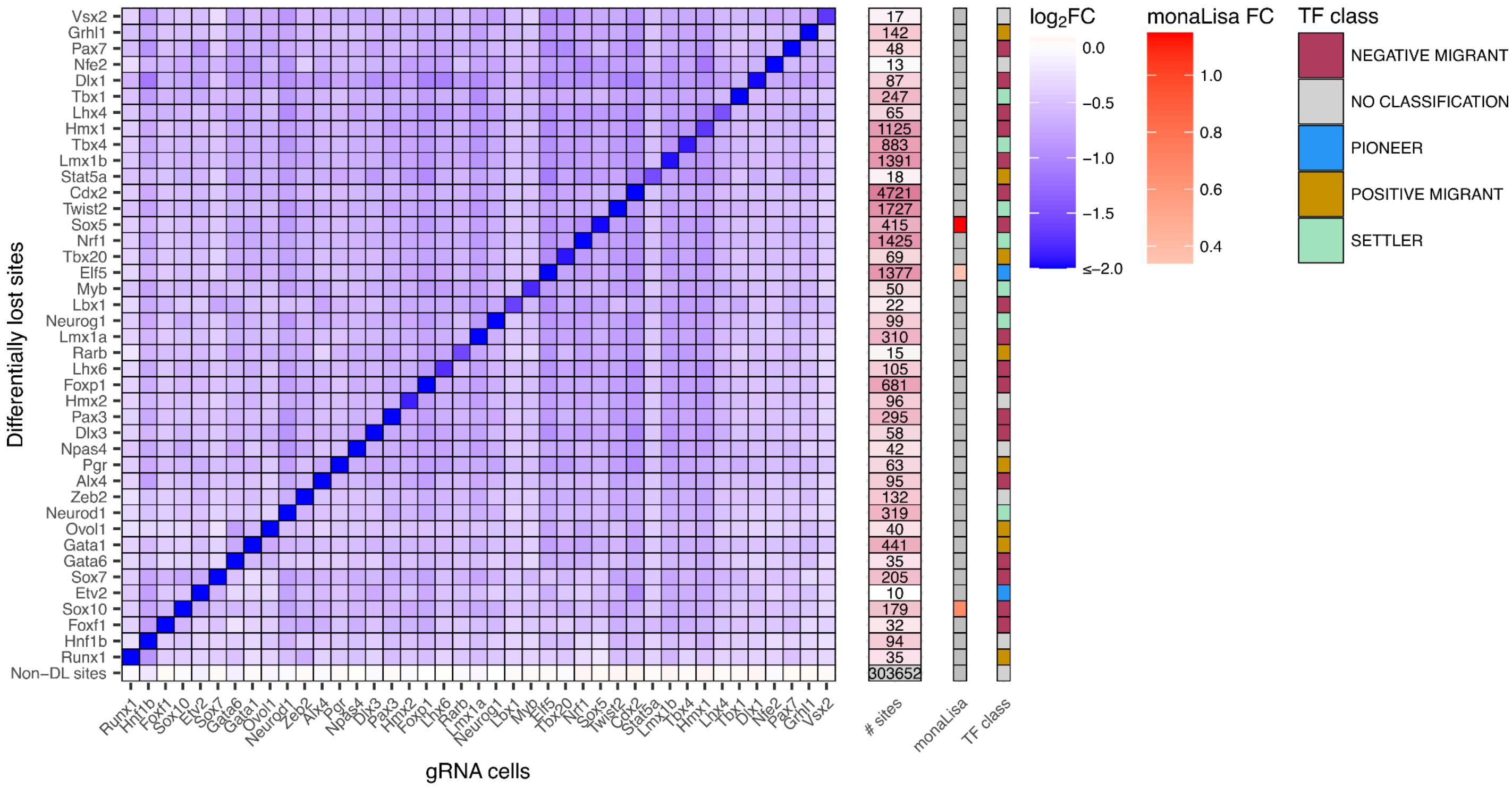

Figure S8. Overlap in chromatin accessibility changes at differentially lost sites between TFs.
Heatmap showing the average $\log_2$ fold-change in chromatin accessibility (gRNA vs NTC) at differentially lost (DL) sites defined for each TF (rows) across lochNESS-selected gRNA cell populations (columns). DL sites were called for each TF using an empirical 5% FDR threshold; cross-TF heatmap entries reflect the average $\log_2$ fold-change at those sites, without reapplying statistical significance criteria; $\log_2$ fold-change values were capped at ±3 for visualization. TFs (rows) were hierarchically clustered (Ward.D2) based on their DL-site accessibility change profiles across gRNA cell populations. Only TFs with at least 10 DL sites are visualized. Non-DL sites (top row) correspond to the average $\log_2$ fold-change across all peaks in the dataset that were not called DL for any TF. The adjacent vertical tile panels indicate, from left to right, the number of DL sites contributing to each TF's row, the enrichment of the matching TF motif family within DL sites calculated using monaLisa with GC-matched genomic background sequences (most enriched motif from the matching TF family with $-\log_{10}$ adjusted $p > 2$), and the chromatin-opening classification assigned to each TF by Ehsani et al.

bioRxiv preprint doi: https://doi.org/10.64898/2026.10.07.757445; this version posted October 9, 2026. The copyright holder for this preprint (which was not certified by peer review) is the author/funder, who has granted bioRxiv a license to display the preprint in perpetuity. It is made available under a CC-BY 4.0 International license.

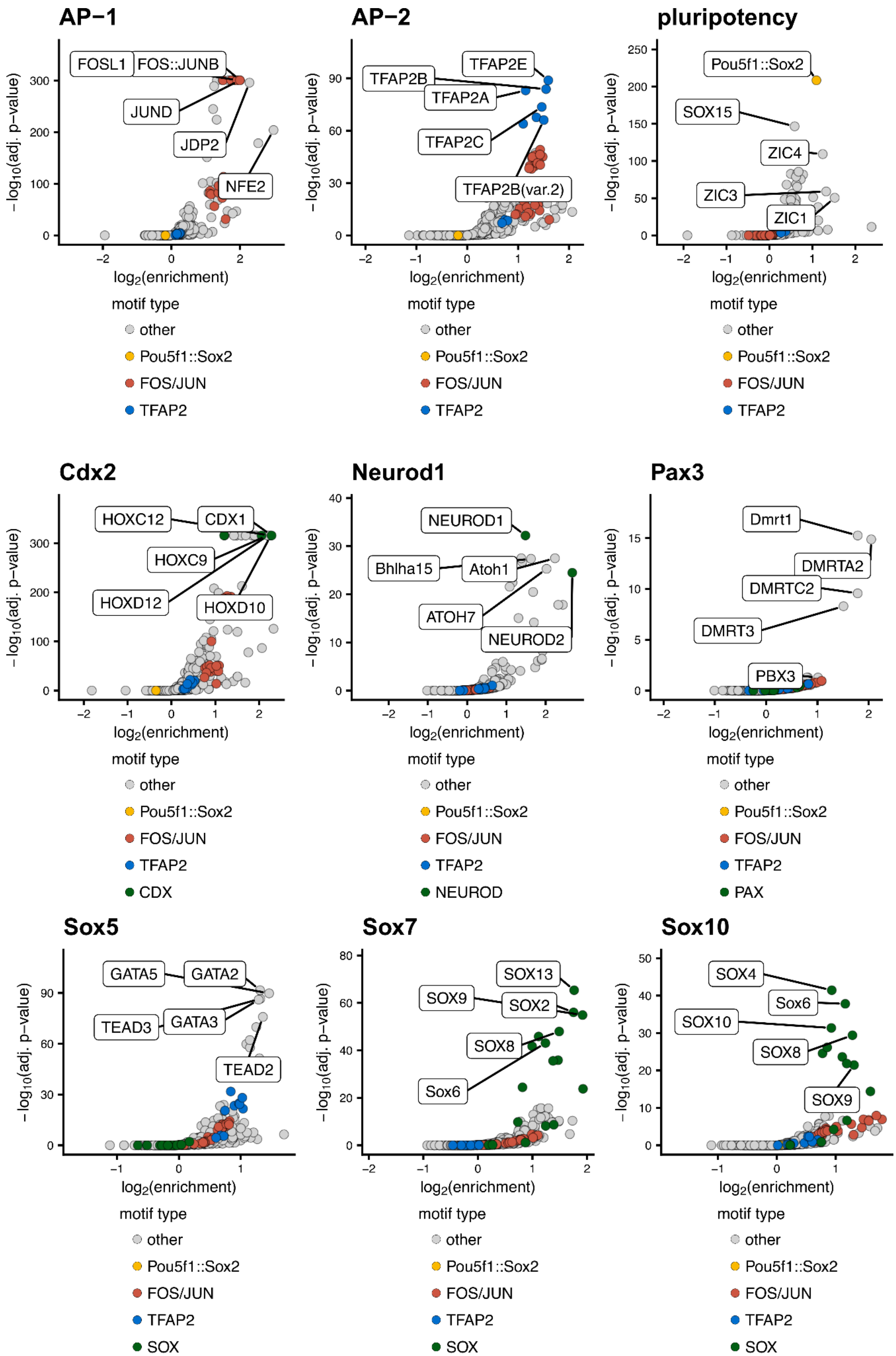

bioRxiv preprint doi: https://doi.org/10.64898/2026.10.07.757445; this version posted October 9, 2026. The copyright holder for this preprint (which was not certified by peer review) is the author/funder, who has granted bioRxiv a license to display the preprint in perpetuity. It is made available under a CC-BY 4.0 International license.

Figure S9. Motif enrichment in DGA peak modules

Motif enrichment in different groups of DGA sites; (i) TF-specific groups (*Cdx2*, *Sox7*, *Neurod1*, *Sox10*, *Pax3*, and *Sox5*), comprising sites called as DG exclusively for the indicated TF (for *Sox5*, including sites DG for *Sox5* and/or *Nrf1* but no other TF); (ii) AP-2, comprising sites DG for one or more trophoblast-associated TFs (*Elf5*, *Grhl1*, *Gata1*, *Gata5*, Gata6, Ovol1) and no other TFs; (iii) AP-1, comprising remaining DG sites not assigned to the above categories; and (iv) pluripotency, comprising sites called as DL for any TF and not called as DG for any TF. For each of the above groups, motif enrichment was performed with MonaLisa using GC-matched genomic background sequences. Volcano plots show the $\log_2$(enrichment) of all motifs on the x-axis and $-\log_{10}$(adjusted p-value) on the y-axis. In the color legend, motifs are classified as “FOS/JUN” if the motif ID contains “Fos” or “Jun,” as “TFAP2” if the motif ID contains “TFAP2,” as “Pou5f1::Sox2” for that specific motif, and as “other” for all remaining motifs. For TF-specific groups, motifs whose IDs matched the target TF family name (e.g., NEUROD for Neurod1, SOX for Sox7, etc.) were additionally assigned to a TF-family–specific category and colored separately. The top 10 motifs with the largest combined enrichment and significance score ($\log_2$(enrichment) x $-\log_{10}$(adjusted p-value)) are labeled.

bioRxiv preprint doi: https://doi.org/10.64898/2026.10.07.757445; this version posted October 9, 2026. The copyright holder for this preprint (which was not certified by peer review) is the author/funder, who has granted bioRxiv a license to display the preprint in perpetuity. It is made available under a CC-BY 4.0 International license.

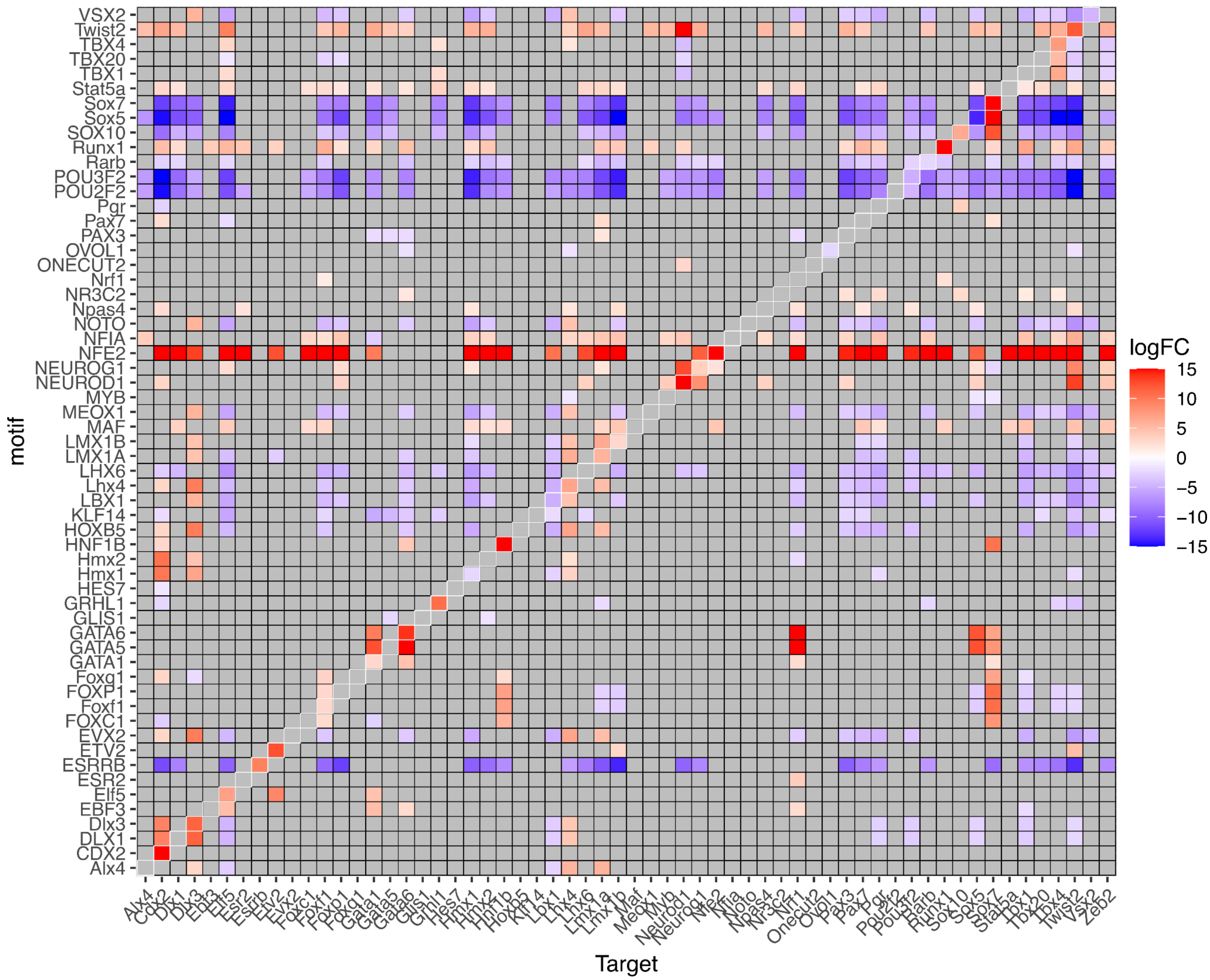

Figure S10. Target TF motif accessibility analysis with chromVAR

Heatmap showing the fold change in chromVAR-derived motif accessibility in target TF gRNA(+) cells (columns) relative to NTC gRNA(+) cells for each TF's motif (rows). Tiles are colored by $\log_2$ fold change, with values corresponding to adjusted $p > 0.05$ shown in grey. White boxes along the diagonal highlight each TF's own motif ($n = 21$ passing significance and logFC > 0).

bioRxiv preprint doi: https://doi.org/10.64898/2026.10.07.757445; this version posted October 9, 2026. The copyright holder for this preprint (which was not certified by peer review) is the author/funder, who has granted bioRxiv a license to display the preprint in perpetuity. It is made available under a CC-BY 4.0 International license.

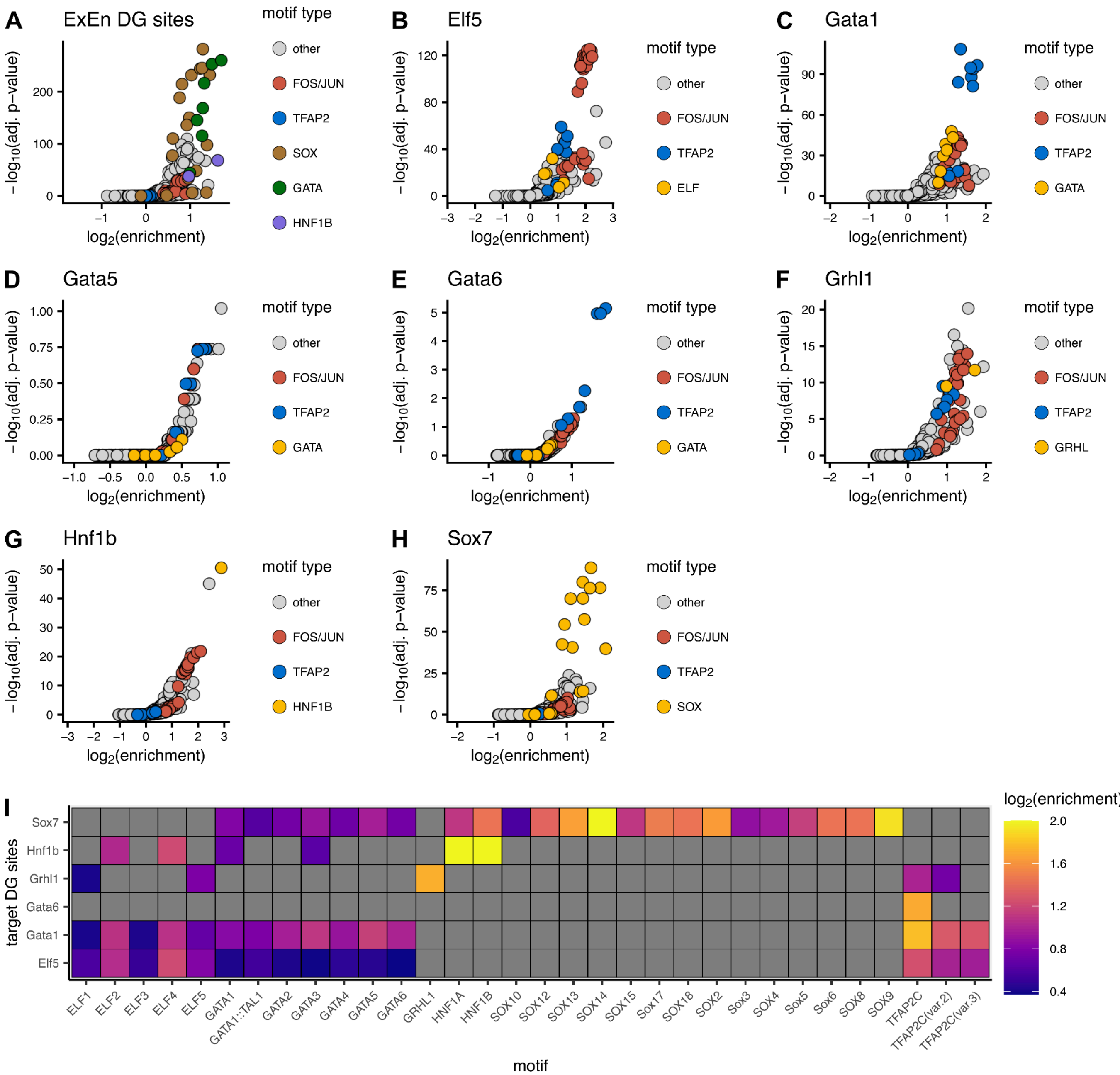

Figure S11. Motif enrichment of target TF motifs and the TFAP2C motif in TF DG sites

(A) Motif enrichment in DG sites called on all cells within the ExEn annotated ATAC cluster; motif enrichment was performed with MonaLisa using GC-matched genomic background sequences. **(B)-(H)** For each of the indicated TF's DG sites, motif enrichment was performed with MonaLisa using GC-matched genomic background sequences. Volcano plots show the $\log_2$(enrichment) of all motifs on the x-axis and -$\log_{10}$(adjusted p-value) on the y-axis. In the color legend, motifs are classified as "FOS/JUN" if the motif ID contains "Fos" or "Jun," as "TFAP2" if the motif ID contains "TFAP2", as the TF-specific family (e.g. GATA, SOX) for motifs matching the indicated TF, and as "other" for all remaining motifs. **(I)** Heatmap showing motif enrichment (as described above in **B-H**) for the motif families ELF, GATA, GRHL1, HNF1, SOX, and TFAP2C in the target DG sites of *Elf5*, *Gata1*, *Gata6*, *Grhl1*, *Hnf1b*, and *Sox7* cells, indicating shared enrichment of TFAP2C among *Elf5*, *Gata1*, *Gata6*, and *Grhl1.*

bioRxiv preprint doi: https://doi.org/10.64898/2026.10.07.757445; this version posted October 9, 2026. The copyright holder for this preprint (which was not certified by peer review) is the author/funder, who has granted bioRxiv a license to display the preprint in perpetuity. It is made available under a CC-BY 4.0 International license.

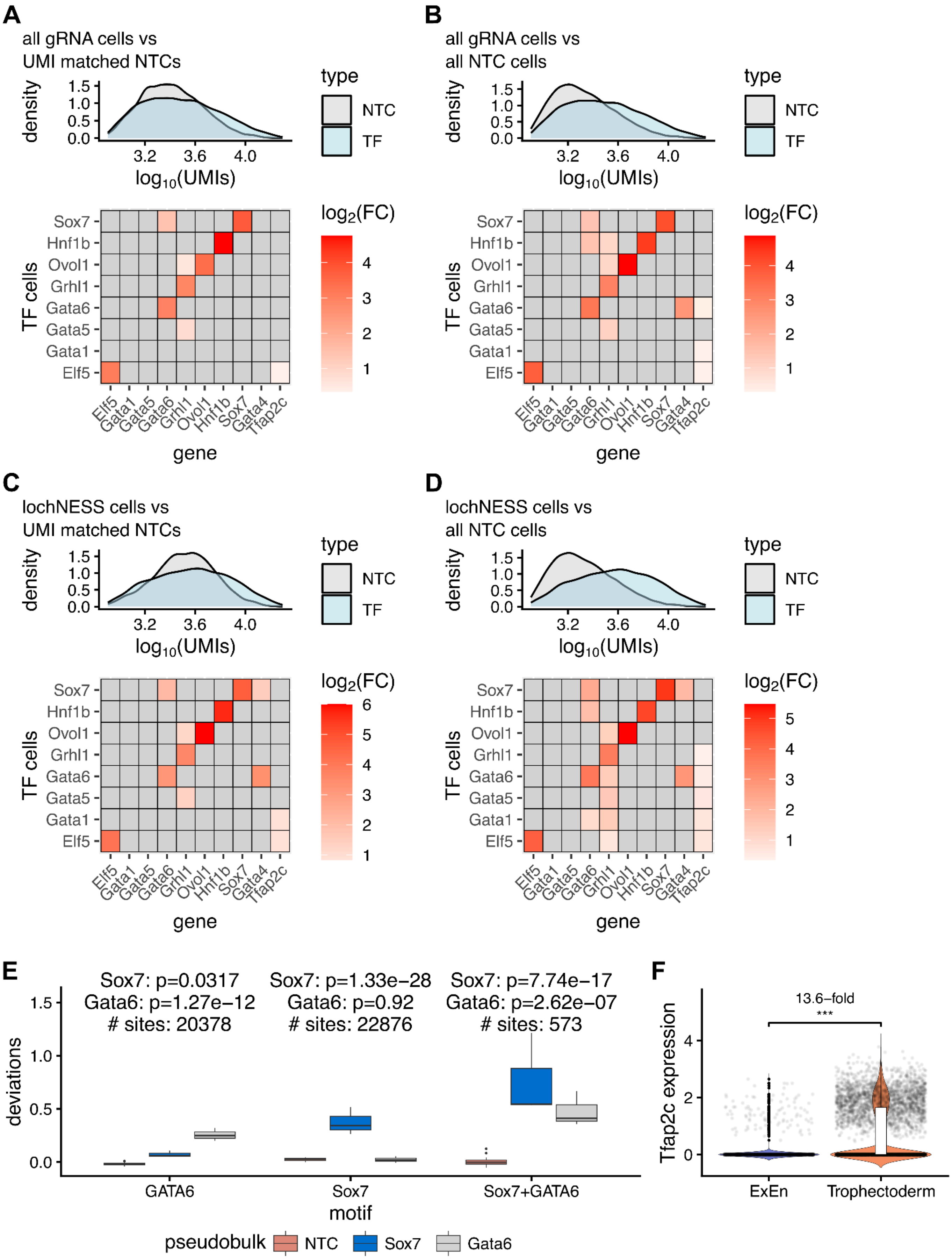

bioRxiv preprint doi: https://doi.org/10.64898/2026.10.07.757445; this version posted October 9, 2026. The copyright holder for this preprint (which was not certified by peer review) is the author/funder, who has granted bioRxiv a license to display the preprint in perpetuity. It is made available under a CC-BY 4.0 International license.

Figure S12. Differential expression analysis comparing TF cells to NTC cells under different cell selection strategies, showing overall consistent effects.

Heatmap (lower subpanels) showing differential expression of the indicated genes (columns) in TF gRNA(+) cells (rows) compared with NTC cells. The TF gRNA(+) cells used were either all gRNA(+) cells or lochNESS-selected gRNA(+) cells, and the NTC cells used were either all NTC cells or NTC cells subsampled to match the per-cell UMI distribution of TF cells (as indicated in panel titles). Differential expression was tested using a Wilcoxon rank-sum test. Values are shown in grey if they did not pass BH-adjusted $p < 0.01$, had $\log_2 FC < \log_2(1.2)$, or were detected in fewer than 5% of TF cells. The density plots (upper subpanels) show the $\log_{10}$-normalized per-cell UMI distribution across all TF cells used in the respective testing (blue) relative to all of the corresponding NTC cells used for testing (light grey). In all panels/comparisons, *Gata1* and *Gata5* exhibited significant TF activation (≥4-fold increase; BH-adjusted $p < 0.01$) but are shown in grey along the diagonal because the target genes were detected in fewer than 5% of TF cells and therefore did not pass the minimum detection threshold. **(A)** All TF gRNA(+) cells compared with UMI-matched NTC cells. An increase in *Tfap2c* expression passing thresholding was only observed for *Elf5* (1.27-fold) **(B)** All TF gRNA(+) cells compared with all NTC cells. The largest increase in *Tfap2c* expression passing thresholding was observed for *Gata6* (1.31-fold) **(C)** lochNESS-selected TF gRNA(+) cells compared with UMI-matched NTC cells. The largest increase in *Tfap2c* expression passing thresholding was observed for *Gata1* (1.83-fold). **(D)** lochNESS-selected TF gRNA(+) cells compared with all NTC cells. (**E**) Boxplots showing chromVAR deviations for the GATA6 motif, Sox7 motif, and Sox7+GATA6 co-motif instances (in which the motifs are between 5-100bp of one another) for Gata6, Sox7, and NTC pseudobulked cells. The adjusted p-values between Sox7 and NTC cells as well as between Gata6 and NTC cells are indicated above each pair of boxplots, as well as the number of sites / motif instances used for the analysis. (**F**) Violin plot showing *Tfap2c* expression in cells from the ExEn-like cluster (left) and trophectoderm-like cluster (right). Fold change and significance were calculated by comparing cells between the two clusters.

bioRxiv preprint doi: https://doi.org/10.64898/2026.10.07.757445; this version posted October 9, 2026. The copyright holder for this preprint (which was not certified by peer review) is the author/funder, who has granted bioRxiv a license to display the preprint in perpetuity. It is made available under a CC-BY 4.0 International license.

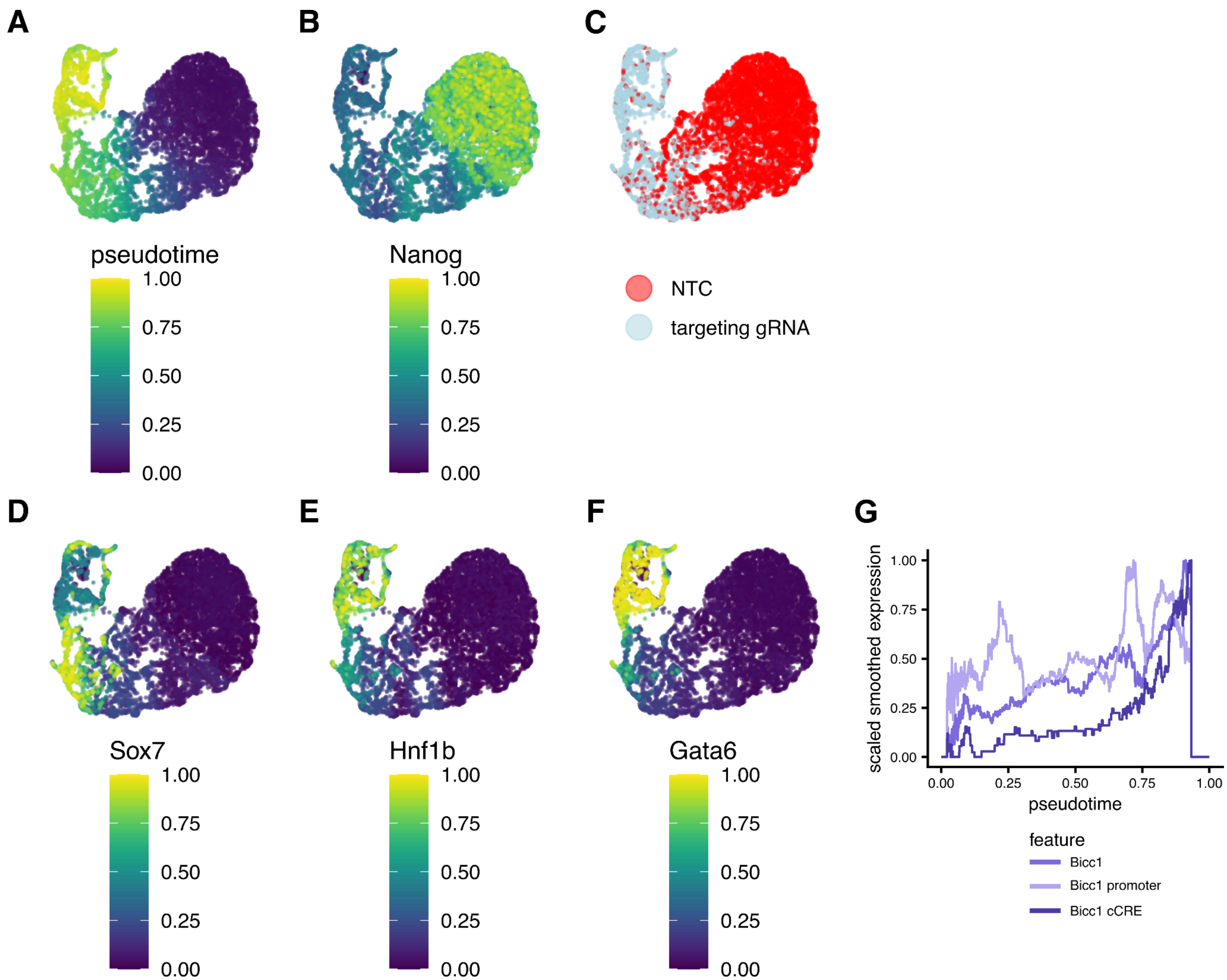

Figure S13. Pseudotime ordering of cells within the ExEn-like state
(**A**) UMAP visualization of the diffusion pseudotime embedding constructed using all cells from the ExEn-like cluster together with NTC gRNA(+) cells. Pseudotime values inferred from the diffusion map are overlaid onto the embedding. (**B**) UMAP visualizations showing smoothed expression of *Nanog* across the pseudotime embedding. Expression values were smoothed across neighboring cells ordered by pseudotime and scaled from 0–1 independently for each feature. (**C**) UMAP visualization showing the distribution of NTC gRNA(+) cells versus all targeting gRNA(+) cells across the pseudotime embedding. NTC cells are concentrated in the earliest pseudotime region corresponding to the *Nanog*-high pluripotent state, whereas targeting gRNA(+) cells preferentially occupy later regions of the trajectory. **(D)–(F)** Smoothed expression profiles for *Sox7* (**D**), *Hnf1b* (**E**), and *Gata6* (**F**) across pseudotime. Expression values were smoothed with a rolling average and independently scaled from 0–1 for visualization. **(G)** Smoothed expression and accessibility profiles of the gene *Bicc1*, a peak overlapping the *Bicc1* promoter, and a peak intronic to *Bicc1* ("*Bicc1* cCRE") across pseudotime.

bioRxiv preprint doi: https://doi.org/10.64898/2026.10.07.757445; this version posted October 9, 2026. The copyright holder for this preprint (which was not certified by peer review) is the author/funder, who has granted bioRxiv a license to display the preprint in perpetuity. It is made available under a CC-BY 4.0 International license.

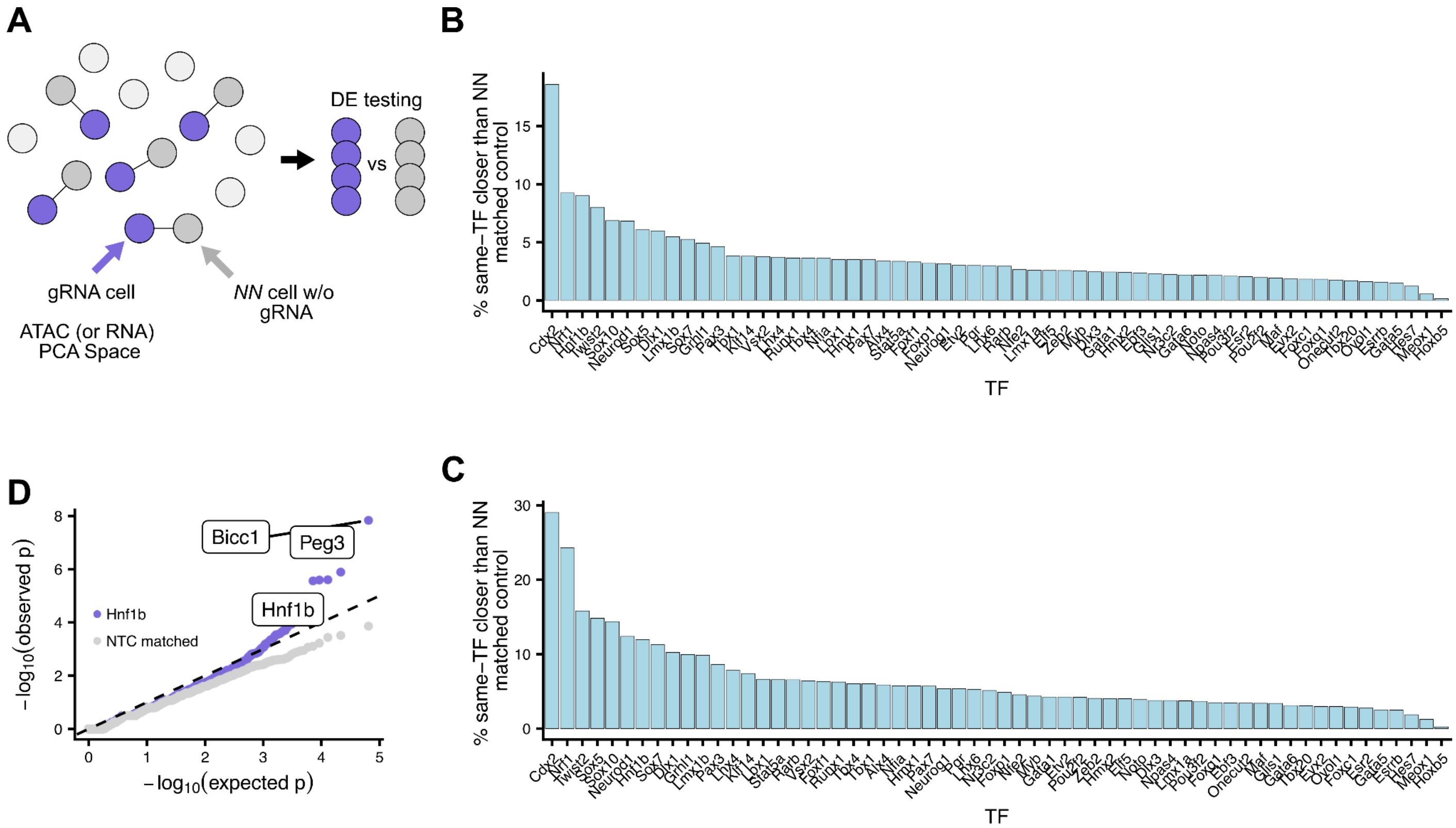

Figure S14. Assessment of nearest-neighbor (NN) matching distance in PCA space across TF perturbations. **(A)** Schematic representation of NN approach. For each gRNA cell the NN cell in PC space is identified and used as out group for differential expression (or accessibility) testing. **(B)** For each TF, we quantified the percentage of all gRNA-assigned cells for which the nearest cell carrying the same TF perturbation was closer in PCA space than the matched control selected for NN differential expression testing. For each target cell, the matched control was defined as the nearest neighbor not assigned that TF and not previously selected as a control for another cell. Bars represent the percentage of cells per TF where the nearest TF cell was closer than the matched control cell. **(C)** Same as in **B** only using lochNESS-selected gRNA(+) cells instead of all gRNA(+) cells. **(D)** Quantile-quantile plot showing distribution of expected vs observed p-values from running the NN DE testing on *Hnf1b* gRNA(+) cells (purple dot) or creating a null distribution by performing the same analysis using a matched number of NTC cells (grey dots).

bioRxiv preprint doi: https://doi.org/10.64898/2026.10.07.757445; this version posted October 9, 2026. The copyright holder for this preprint (which was not certified by peer review) is the author/funder, who has granted bioRxiv a license to display the preprint in perpetuity. It is made available under a CC-BY 4.0 International license.

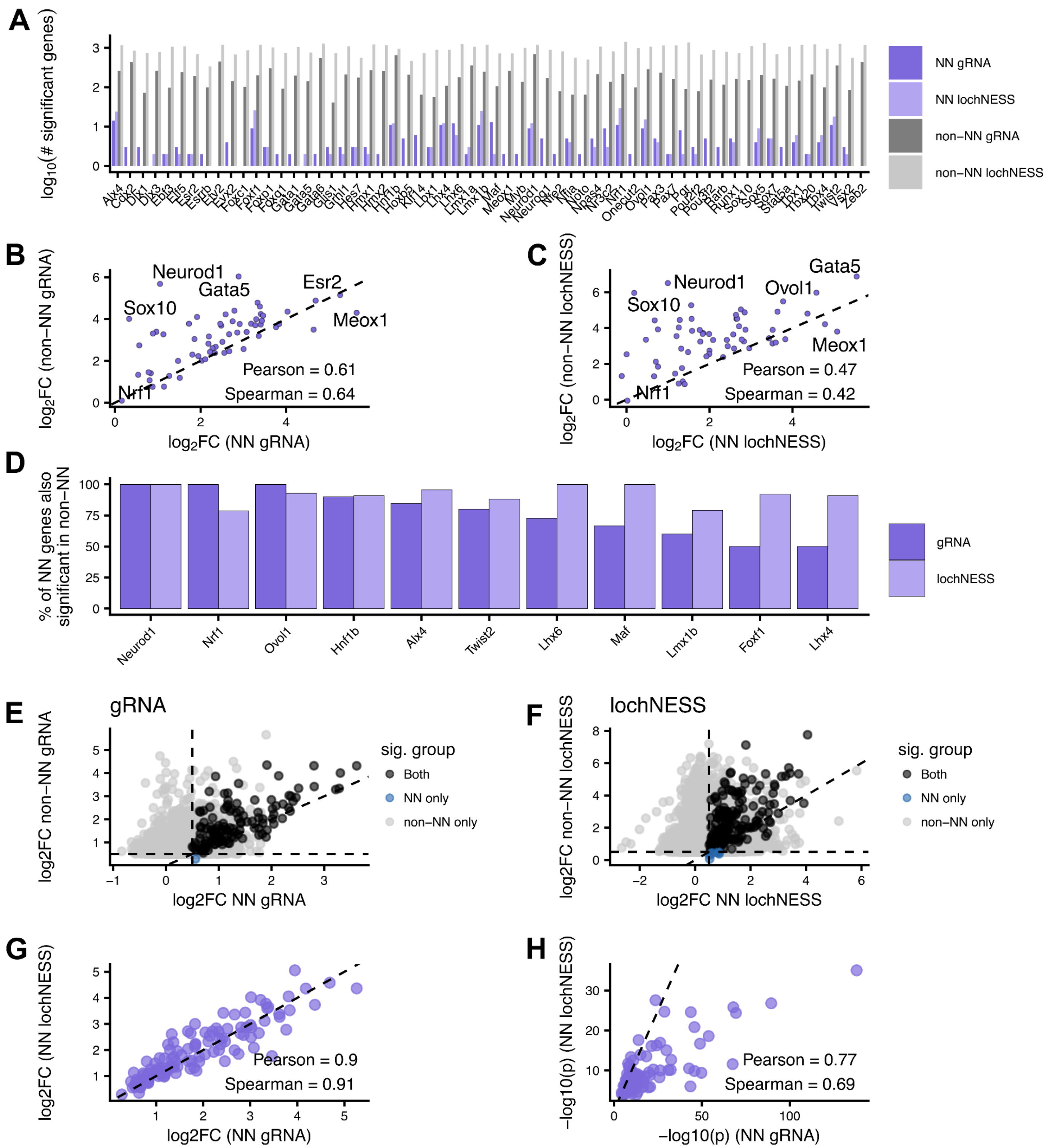

Figure S15. Comparison of nearest-neighbor (NN) differential expression and standard differential expression across TF perturbations.

(A) Number of significant DE genes identified per TF by NN-DE using all gRNA(+) cells (dark purple), NN-DE using lochNESS-selected gRNA(+) cells (light purple), standard DE using all gRNA(+) cells (dark grey), and standard DE using all lochNESS-selected gRNA(+) cells (light grey). Genes were considered differentially expressed if they passed a 10% empirical FDR threshold derived from NTC-vs-NTC matched comparisons, exhibited positive effect size (log_2FC >

bioRxiv preprint doi: https://doi.org/10.64898/2026.10.07.757445; this version posted October 9, 2026. The copyright holder for this preprint (which was not certified by peer review) is the author/funder, who has granted bioRxiv a license to display the preprint in perpetuity. It is made available under a CC-BY 4.0 International license.

0.1 for NN, $\log_2$FC > 1 for standard DE), and were detected in ≥5% of either TF gRNA(+) cells or matched control cells. **(B)** Correlations in target TF activation FC values using NN approach (x-axis) vs standard DE approach (y-axis). All gRNA(+) cells were used for testing. **(C)** Correlations in target TF activation FC values using NN approach (x-axis) vs standard DE approach (y-axis). LochNESS-selected gRNA(+) cells were used for testing. **(D)** Fraction of NN-DE genes that are also identified by standard differential expression (DE) for each TF. Bars are colored by the number of NN-DE genes detected per TF. Only TFs with ≥10 NN-DE genes are shown. (**E**) Scatter plot showing the correlation of NN-DE $\log_2$ fold-change values (x-axis) vs standard DE $\log_2$ fold-change values (y-axis) for all genes called as significant by either method and using all gRNA(+) cells for respective DE testing. (**F**) Same as in **E** only using lochNESS-selected gRNA(+) cells for the respective DE testing. (**G**) Scatter plot showing the correlation of NN-DE $\log_2$ fold-change values using all gRNA(+) cells (x-axis) vs NN-DE $\log_2$ fold-change values using lochNESS-selected gRNA(+) cells for all genes called as significant by either method. (**H**) same as in **G**, only plotting $-\log_{10}$ p-values instead of effect sizes.

bioRxiv preprint doi: https://doi.org/10.64898/2026.10.07.757445; this version posted October 9, 2026. The copyright holder for this preprint (which was not certified by peer review) is the author/funder, who has granted bioRxiv a license to display the preprint in perpetuity. It is made available under a CC-BY 4.0 International license.

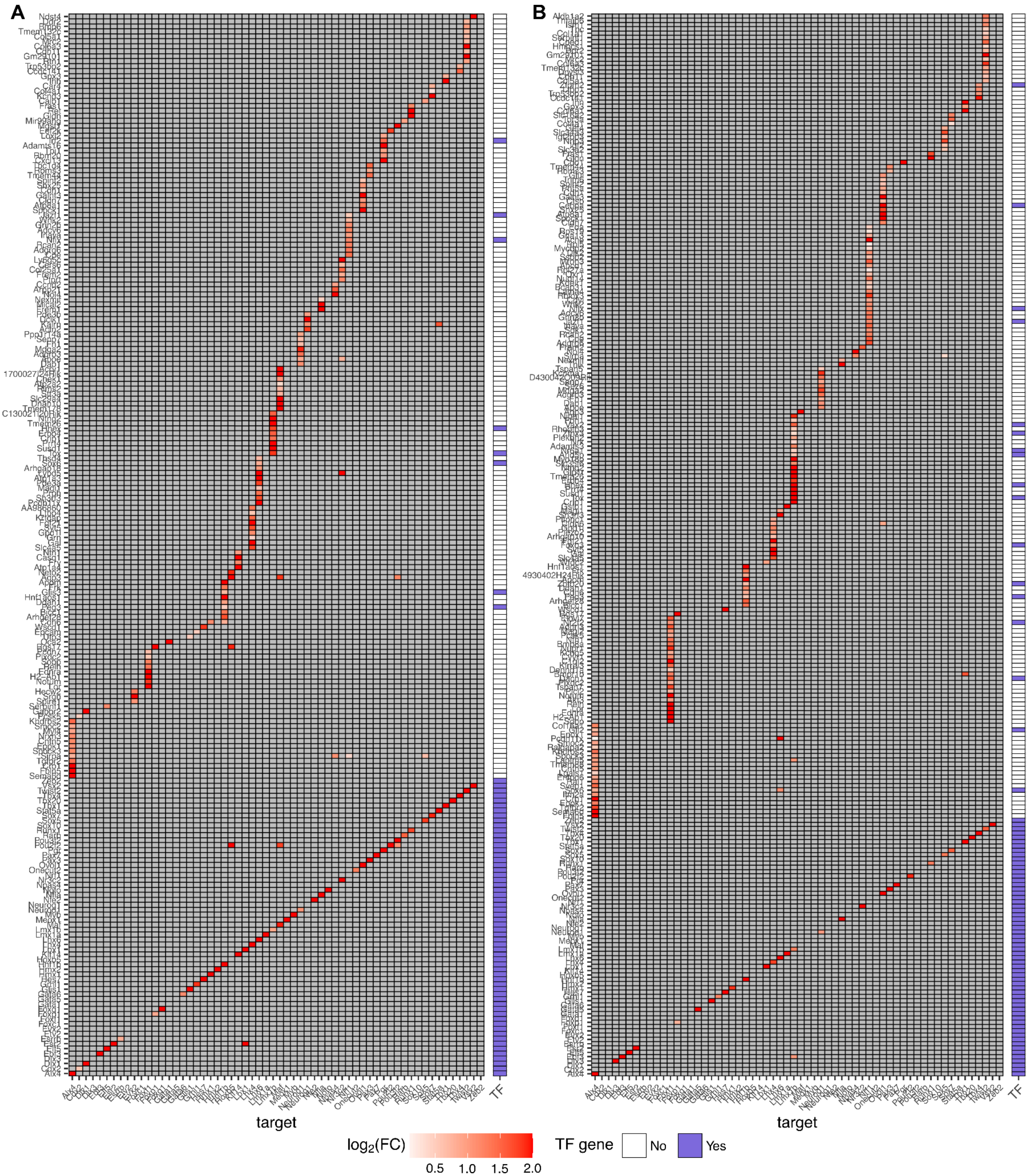

bioRxiv preprint doi: https://doi.org/10.64898/2026.10.07.757445; this version posted October 9, 2026. The copyright holder for this preprint (which was not certified by peer review) is the author/funder, who has granted bioRxiv a license to display the preprint in perpetuity. It is made available under a CC-BY 4.0 International license.

Figure S16. Nearest-neighbor differential expression across all TF perturbations

(A) Heatmap summarizing nearest-neighbor (NN) differential expression analysis for all TF perturbations using all gRNA-assigned cells. For each TF-targeting gRNA, cells were matched one-to-one with the nearest cell in ATAC PCA space that was assigned either a different TF-targeting gRNA or a non-targeting control (NTC) gRNA and had not already been selected as a control. This matching procedure minimizes differences arising from pre-existing chromatin state heterogeneity while excluding cells assigned the same perturbation. Differential expression was then performed between each TF perturbation and its matched controls. Statistical significance was determined using an empirical false discovery rate (FDR) framework in which the NN differential expression analysis was repeated five times using matched NTC cells to generate an empirical null distribution of p-values. Empirical p-values were calculated as the fraction of null p-values at least as significant as the observed p-value, and an empirical FDR threshold of 10% was determined for each TF using the Benjamini–Hochberg procedure applied to these empirical p-values. Genes with average $\log_2$ fold change >0.1 passing the corresponding empirical FDR threshold are shown in color, whereas non-significant comparisons are shown in gray. Rows include all targeted TFs together with genes that were significantly upregulated for at least one perturbation. Colors indicate average $\log_2$ fold change relative to matched controls, with values greater than 2 truncated at 2. The adjacent binary annotation indicates whether each gene is a curated murine transcription factor. **(B)** Same analysis as in (**A**), except NN differential expression was performed using only the subset of cells assigned to each perturbation and passing lochNESS filtering.

bioRxiv preprint doi: https://doi.org/10.64898/2026.10.07.757445; this version posted October 9, 2026. The copyright holder for this preprint (which was not certified by peer review) is the author/funder, who has granted bioRxiv a license to display the preprint in perpetuity. It is made available under a CC-BY 4.0 International license.

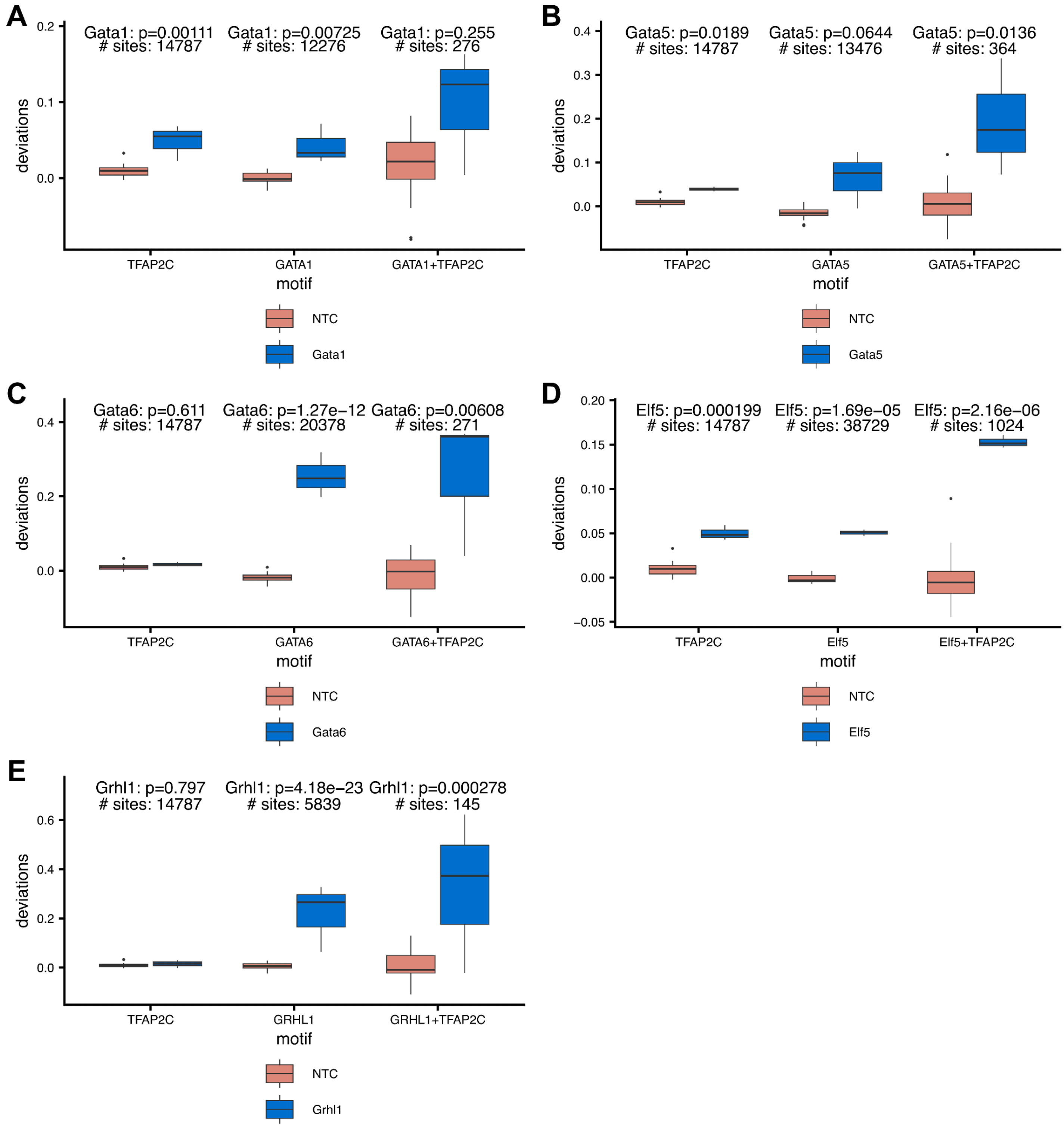

Figure S17. Increase in co-motif accessibility for different TF targets.
(A) Boxplots showing chromVAR deviations for the TFAP2C motif, GATA1 motif, and GATA1+TFAP2C co-motif instances (in which the motifs are between 5-100bp of one another) for *Gata1* and NTC pseudobulked cells. The significance between *Gata1* and NTC cells is indicated above each pair of boxplots, as well as the number of sites / motif instances used for the analysis. **(B)–(E)** Same as (**A**), shown for the indicated TF and its corresponding motif and TFAP2C co-motif classes.

bioRxiv preprint doi: https://doi.org/10.64898/2026.10.07.757445; this version posted October 9, 2026. The copyright holder for this preprint (which was not certified by peer review) is the author/funder, who has granted bioRxiv a license to display the preprint in perpetuity. It is made available under a CC-BY 4.0 International license.

bioRxiv preprint doi: https://doi.org/10.64898/2026.10.07.757445; this version posted October 9, 2026. The copyright holder for this preprint (which was not certified by peer review) is the author/funder, who has granted bioRxiv a license to display the preprint in perpetuity. It is made available under a CC-BY 4.0 International license.

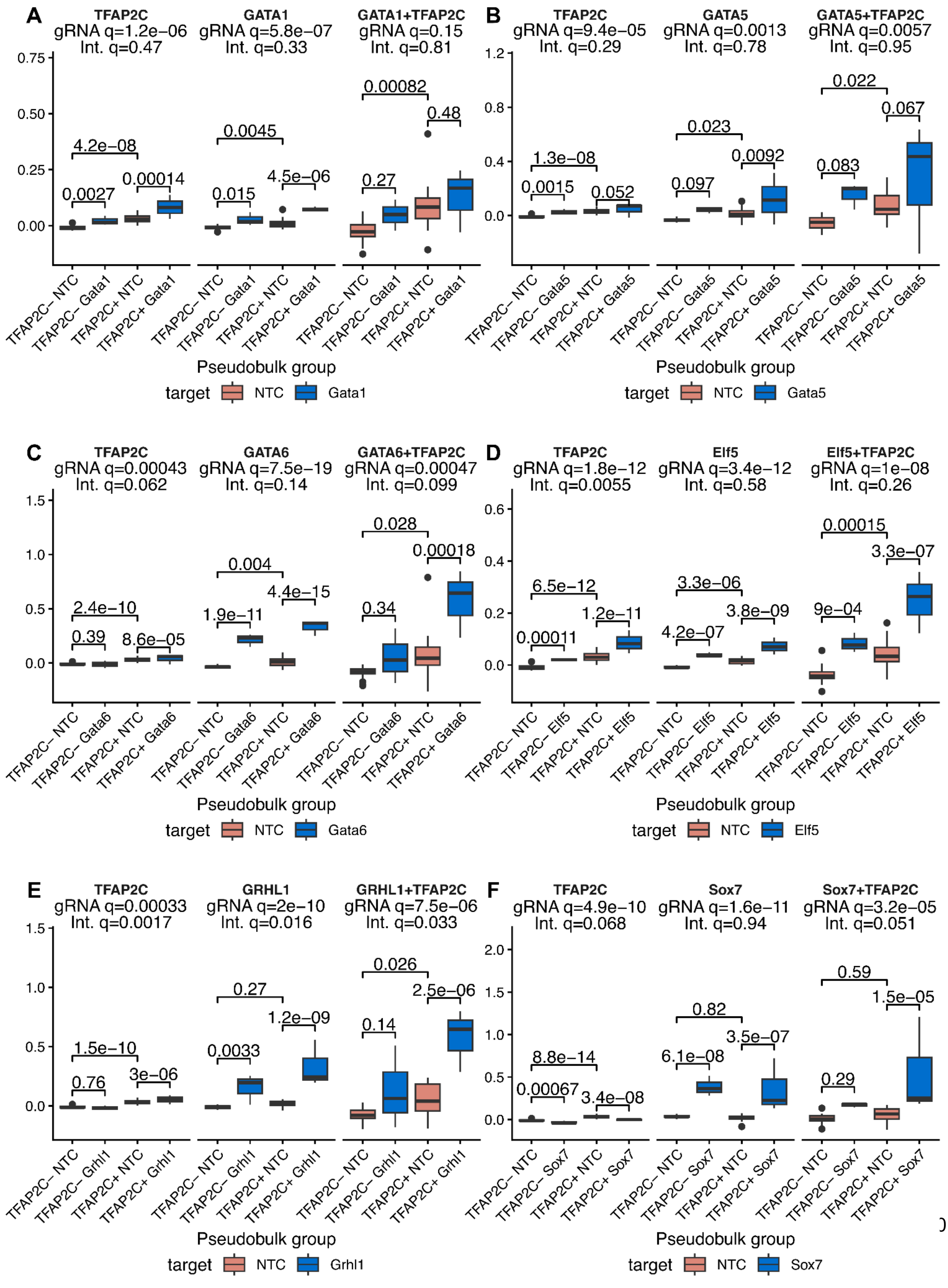

bioRxiv preprint doi: https://doi.org/10.64898/2026.10.07.757445; this version posted October 9, 2026. The copyright holder for this preprint (which was not certified by peer review) is the author/funder, who has granted bioRxiv a license to display the preprint in perpetuity. It is made available under a CC-BY 4.0 International license.

Figure S18. Co-motif enrichment for different TF targets after stratifying by *Tfap2c* expression (single gRNA assigned cells only).

(A) Boxplots showing chromVAR deviation scores at TFAP2C motif sites, GATA1 motif sites, and TFAP2C+GATA1 co-motif sites (motifs located within 5–100 bp of each other) in pseudobulked *Gata1* gRNA and NTC cells, stratified by detection of *Tfap2c* expression (TFAP2C+ and TFAP2C− cells). Adjusted p-values for between-group comparisons are shown. Above each motif class, the significance of the marginal ("global") CRISPRa effect averaged across TFAP2C strata is indicated ("gRNA q ="), along with the significance of the CRISPRa × TFAP2C interaction from a limma model ("Int. q ="). **(B)–(F)** Same as (**A**), shown for the indicated TF and its corresponding motif and TFAP2C co-motif classes.

bioRxiv preprint doi: https://doi.org/10.64898/2026.10.07.757445; this version posted October 9, 2026. The copyright holder for this preprint (which was not certified by peer review) is the author/funder, who has granted bioRxiv a license to display the preprint in perpetuity. It is made available under a CC-BY 4.0 International license.

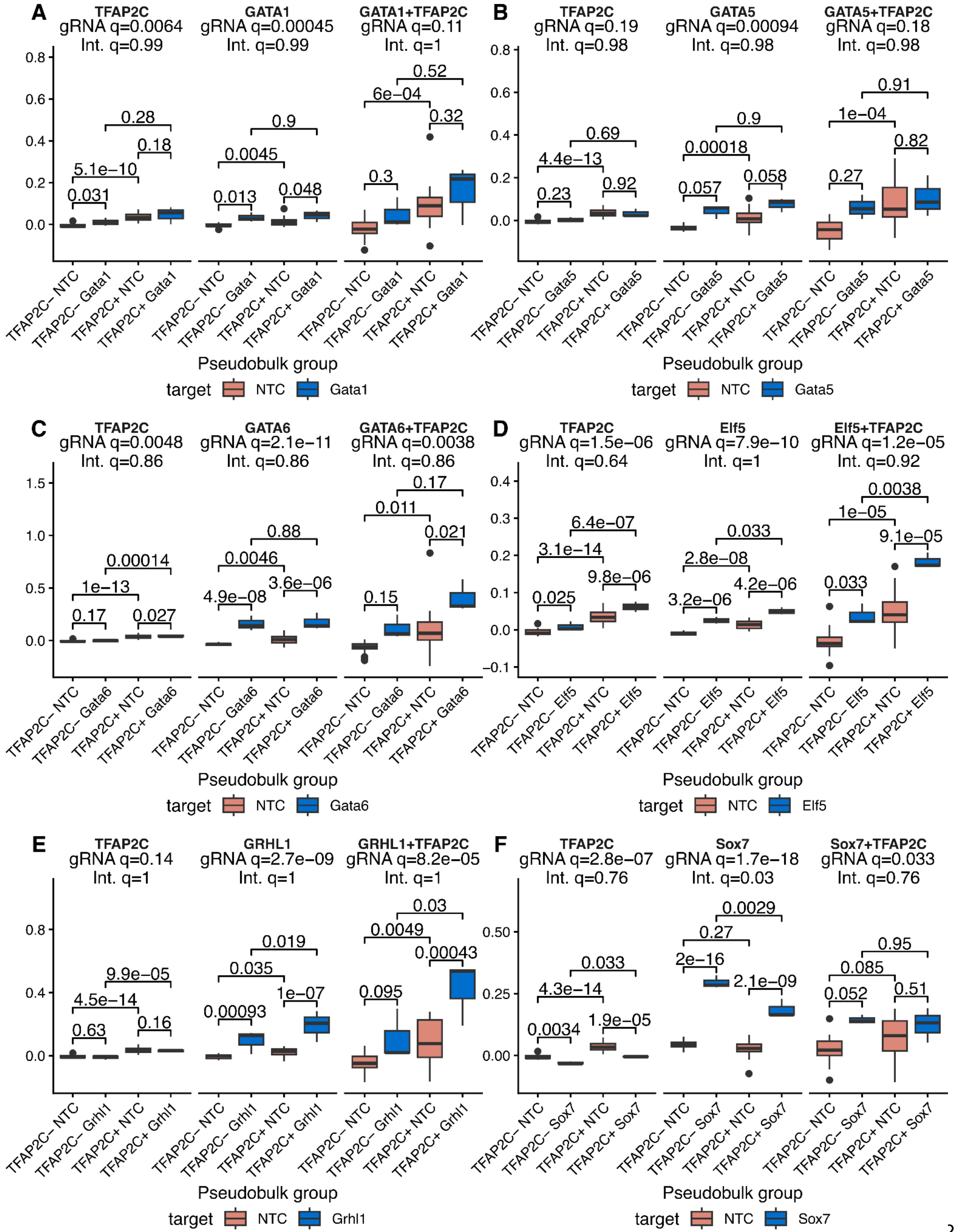

bioRxiv preprint doi: https://doi.org/10.64898/2026.10.07.757445; this version posted October 9, 2026. The copyright holder for this preprint (which was not certified by peer review) is the author/funder, who has granted bioRxiv a license to display the preprint in perpetuity. It is made available under a CC-BY 4.0 International license.

Figure S19. **Co-motif enrichment for different TF targets after stratifying by *Tfap2c* expression (including cells with multiple gRNAs assigned).**

(A) Boxplots showing chromVAR deviation scores at TFAP2C motif sites, GATA1 motif sites, and TFAP2C+GATA1 co-motif sites (motifs located within 5–100 bp of each other) in pseudobulked *Gata1* gRNA and NTC cells, stratified by detection of *Tfap2c* expression (TFAP2C+ and TFAP2C− cells). Adjusted p-values for between-group comparisons are shown. Above each motif class, the significance of the marginal ("global") CRISPRa effect averaged across TFAP2C strata is indicated ("gRNA q ="), along with the significance of the CRISPRa × TFAP2C interaction from a limma model ("Int. q ="). **(B)–(F)** Same as (**A**), shown for the indicated TF and its corresponding motif and TFAP2C co-motif classes.

bioRxiv preprint doi: https://doi.org/10.64898/2026.10.07.757445; this version posted October 9, 2026. The copyright holder for this preprint (which was not certified by peer review) is the author/funder, who has granted bioRxiv a license to display the preprint in perpetuity. It is made available under a CC-BY 4.0 International license.

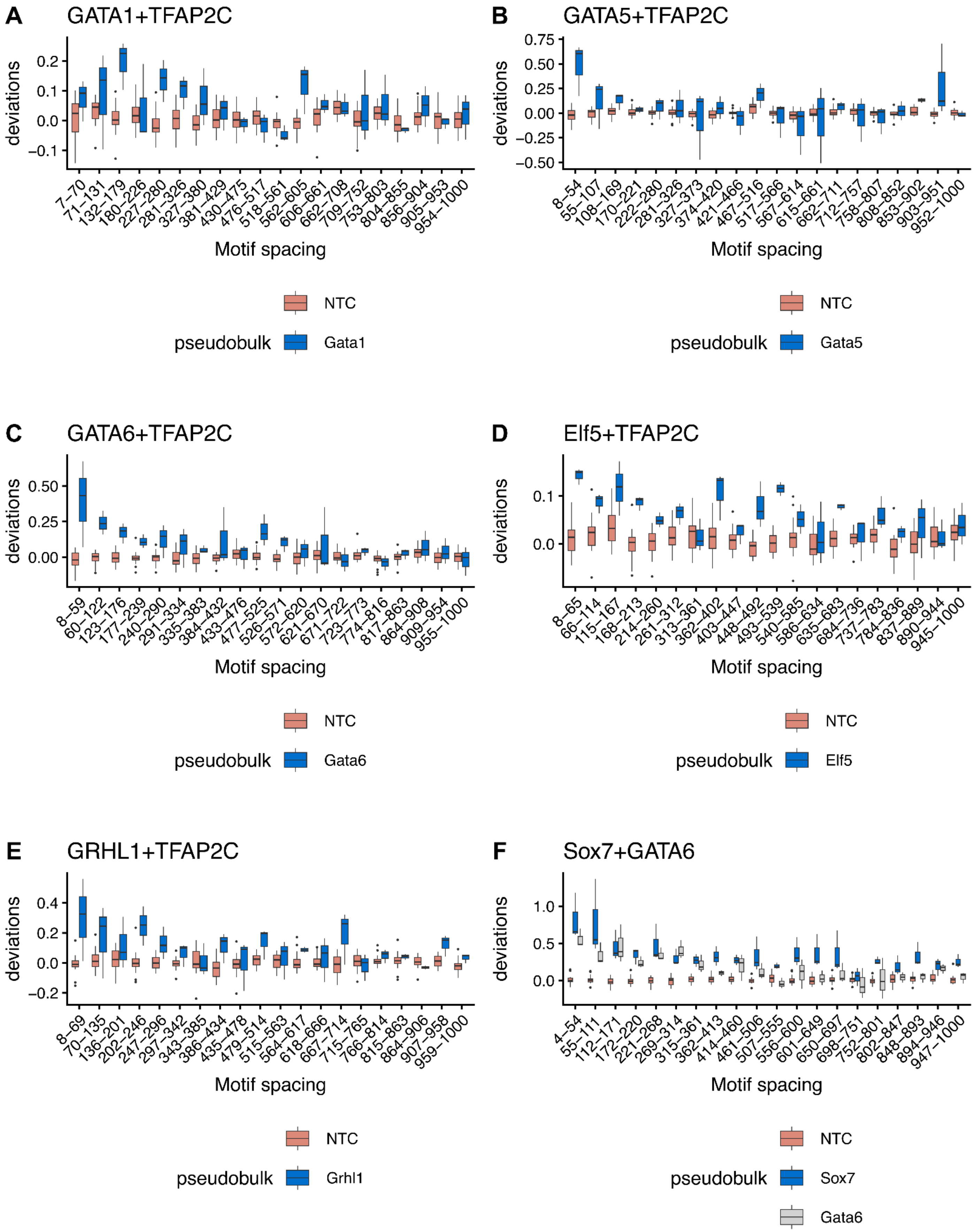

bioRxiv preprint doi: https://doi.org/10.64898/2026.10.07.757445; this version posted October 9, 2026. The copyright holder for this preprint (which was not certified by peer review) is the author/funder, who has granted bioRxiv a license to display the preprint in perpetuity. It is made available under a CC-BY 4.0 International license.

Figure S20. **Co-motif enrichment for different TF targets across varying motif distance bins.**
(A)–(F) chromVAR deviation scores for genomic regions containing pairs of motifs, stratified by the distance between motif instances. For each transcription factor (TF) perturbation, accessibility deviations are shown for regions containing both motifs binned by inter-motif distance (x-axis; bins defined by quantiles of genomic distances between motif pairs, with bin ranges indicated). Boxplots display the distribution of deviation scores across pseudobulked cells for each condition, including TF-targeting gRNA(+) cells and non-targeting controls (NTC). Panels correspond to the following motif pairs: (**A**) The GATA1 + TFAP2C co-motif in *Gata1* gRNA(+) cells, (**B**) The GATA5 + TFAP2C co-motif in *Gata5* gRNA(+) cells, (**C**) The GATA6 + TFAP2C co-motif in *Gata6* gRNA(+) cells, (**D**) The ELF5 + TFAP2C co-motif in *Elf5* gRNA(+) cells, (**E**) The GRHL1 + TFAP2C co-motif in *Grhl1* gRNA(+) cells, and (**F**) The SOX7 + GATA6 co-motif in *Sox7* (blue) and *Gata6* (grey) gRNA(+) cells.

bioRxiv preprint doi: https://doi.org/10.64898/2026.10.07.757445; this version posted October 9, 2026. The copyright holder for this preprint (which was not certified by peer review) is the author/funder, who has granted bioRxiv a license to display the preprint in perpetuity. It is made available under a CC-BY 4.0 International license.

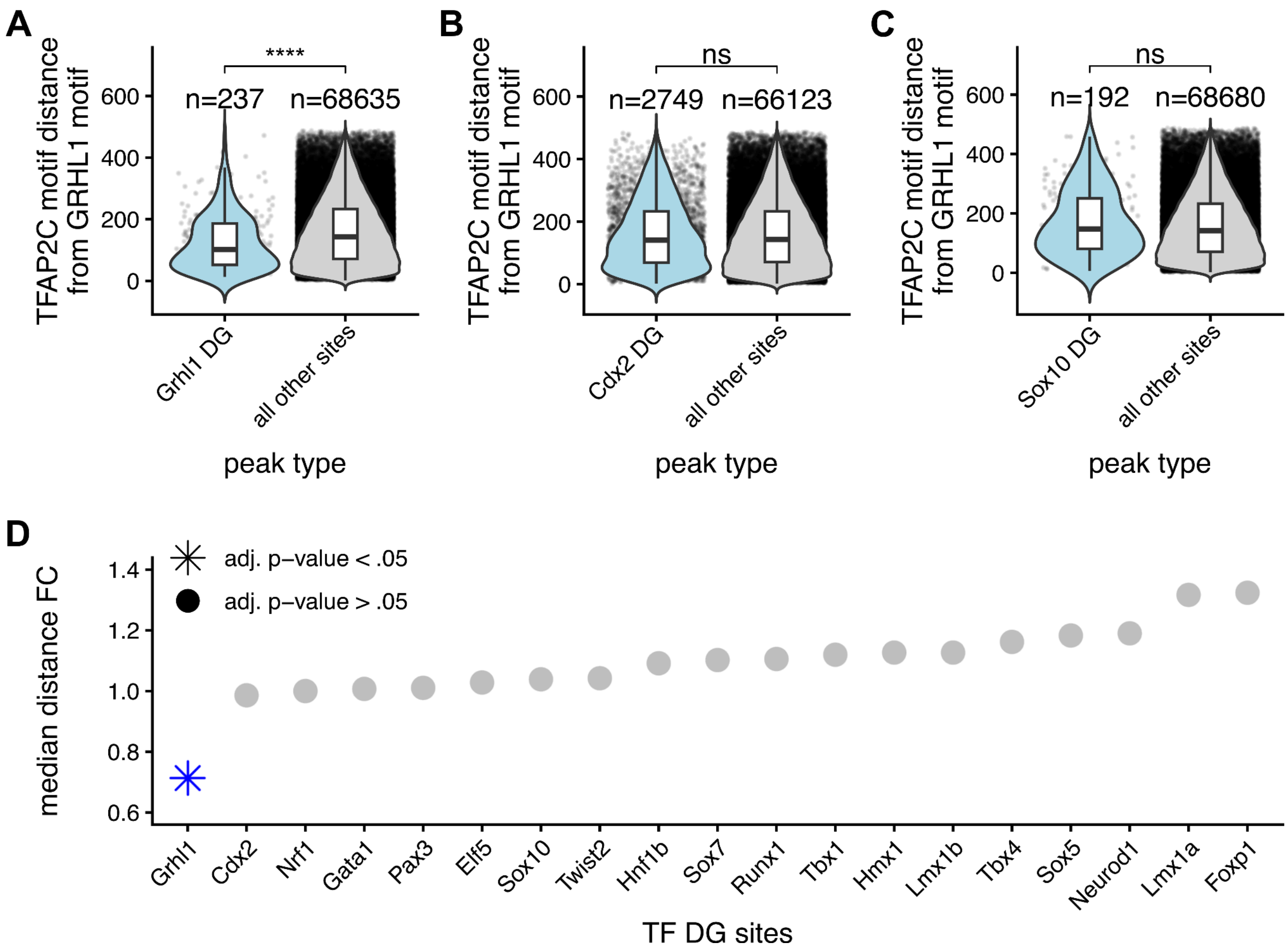

Figure S21. Decreased motif spacing between TFAP2C and GRHL1 motifs in differentially gained sites.
(A) Motif spacing analysis between the TFAP2C motif (JASPAR MA0524.3) and the GRHL1 motif (JASPAR MA0647.2). All ATAC peaks were scanned using FIMO, retaining only the highest-scoring motif instance per peak for each motif (minimum FIMO p-value per peak). Peaks containing both motifs were identified, and the distance between motif midpoints was calculated. Violin plots show the distributions of TFAP2C–GRHL1 motif distances in *Grhl1* differentially gained (DG) peaks (blue violins) versus all other peaks called on the dataset that contain both motifs (grey violins). Numbers above violins indicate the number of peaks containing both motifs per group. Statistical significance was assessed using a two-sided Wilcoxon rank-sum test. **(B)** Same as in (A) only looking at TFAP2C–GRHL1 motif distances in *Cdx2* differentially gained sites, to control for differences being driven by how DG sites are called. **(C)** Same as in (A) only looking at TFAP2C–GRHL1 motif distances in *Sox10* differentially gained sites, to control for differences being driven by how DG sites are called. **(D)** Dot plot showing the fold change in median TFAP2C–GRHL1 motif distance between each TF's DG peaks and all other peaks containing both motifs (median distance in DG sites / median distance in other sites). Only TFs with >50 DG peaks were included. TFs are ordered by FC, and point shape indicates statistical significance after Benjamini–Hochberg multiple testing correction of Wilcoxon p-values.
